# A robust six-gene prognostic signature based on two prognostic subtypes constructed by chromatin regulators is correlated with immunological features and therapeutic response in lung adenocarcinoma

**DOI:** 10.18632/aging.205183

**Published:** 2023-11-07

**Authors:** Qiang Chen, Hongbo Zhao, Jing Hu

**Affiliations:** 1 Faculty of Animal Science and Technology, Yunnan Agricultural University, Kunming, China; 2 Department of Laboratory Animal Science, Kunming Medical University, Kunming, China; 3 Department of Medical Oncology, First People’s Hospital of Yunnan Province, Kunming, China; 4 Department of Medical Oncology, Affiliated Hospital of Kunming University of Science and Technology, Kunming, China

**Keywords:** lung adenocarcinoma, chromatin regulator, risk model, survival, immune cell infiltration

## Abstract

Accumulating evidence has demonstrated that chromatin regulators (CRs) regulate immune cell infiltration and are correlated with prognoses of patients in some cancers. However, the immunological and prognostic roles of CRs in lung adenocarcinoma (LUAD) are still unclear. Here, we systematically revealed the correlations of CRs with immunological features and the survival in LUAD patients based on a cohort of gene expression datasets from the public TCGA and GEO databases and real RNA-seq data by an integrative analysis using a comprehensive bioinformatics method. Totals of 160 differentially expressed CRs (DECRs) were identified between LUAD and normal lung tissues, and two molecular prognostic subtypes (MPSs) were constructed and evaluated based on 27 prognostic DECRs using five independent datasets (*p* =0.016, <0.0001, =0.008, =0.00038 and =0.00055, respectively). Six differentially expressed genes (DEGs) (*CENPK*, *ANGPTL4*, *CCL20*, *CPS1*, *GJB3*, *TPSB2*) between two MPSs had the most important prognostic feature and a six-gene prognostic model was established. LUAD patients in the low-risk subgroup showed a higher overall survival (OS) rate than those in the high-risk subgroup in nine independent datasets (*p* <0.0001, =0.021, =0.016, =0.0099, <0.0001, =0.0045, <0.0001, =0.0038 and =0.00013, respectively). Six-gene prognostic signature had the highest concordance index of 0.673 compared with 19 reported prognostic signatures. The risk score was significantly correlated with immunological features and activities of oncogenic signaling pathways. LUAD patients in the low-risk subgroup benefited more from immunotherapy and were less sensitive to conventional chemotherapy agents. This study provides novel insights into the prognostic and immunological roles of CRs in LUAD.

## INTRODUCTION

Lung cancer (LC), one of the most common malignancies worldwide, was the leading cause of cancer death with 18.0% of total cancer deaths for both sexes combined in 2020 [1]. For men, LC has ranked first in both incidence and mortality, accounting for 14.3% of total new cases and 21.5% of total cancer deaths, respectively [1]. Especially in some countries with a higher human development index (HDI), the incidence and mortality of LC are about 4-fold and 3-fold higher than those in lower HDI countries [1]. The burden of LC incidence and mortality is rapidly growing globally. However, the overall survival (OS) of LC patients remains poor and the overall 5-year survival rate is only about 19% for all stages combined, much lower than 98% of prostate cancer and 90% of female breast cancer [2].

Non-small cell lung cancer (NSCLC) is the most common histological subtype and accounts for about 85% of all LC patients [3], of which lung adenocarcinoma (LUAD) is the major subtype and accounts for greater than 40% of LC cases and its relative frequency is increasing [3, 4]. Currently, the LUAD TNM (tumor-node-metastasis) staging system remains the prevailing method and the most important factor to predict the OS of LUAD patients [5]. However, due to the high heterogeneity of LUAD, the prognoses of LUAD patients within the same TNM group presented heterogeneous outcomes [6], which suggests that some new prognostic methods should be developed and used to refine risk stratification, such as molecular prognostic marker [6]. In recent years, with the progress of molecular biology, some molecular prognostic markers including protein, mRNA, miRNA, lncRNA, and oncogene were identified [6–10]. In addition, liquid biopsies based on circulating tumor cells (CTCs) and circulating free DNA (cfDNA) and tumor microenvironment (TME) based on tumor-infiltrating immune cells (TICs) are of much interest to many scientists in predicting the survival, and some valuable results have been obtained [6, 11, 12].

Epigenetic regulation is an essential mechanism for normal development and maintenance of gene expression patterns in mammals [13]. Growing evidence has shown that epigenetic modification plays a critical role in the regulation of all DNA-based processes, and epigenetic alterations can lead to induction and maintenance of various cancers and are increasingly recognized as a cancer hallmark [14]. Chromatin regulators (CRs) including DNA methylators, histone modifiers and chromatin remodelers, as indispensable regulatory factors, play critical roles in driving epigenetic modification [15]. Some studies have shown that the dysregulation or dysfunction of CRs was correlated with various cancers and some CRs have been used as key drug targets against cancers [16–19]. For example, DNA methylator *DNMT1* has been shown to play an oncogenic role in promoting cell proliferation, migration and invasion by suppressing cell differentiation in pancreatic cancer [20]. Chromatin remodeler *CTCF* has been shown to be upregulated in colorectal cancer tissue, and the overexpression of *CTCF* promoted colorectal cancer cell proliferation [21]. A few recent studies reported that CRs were correlated with the prognoses of cancer patients [22, 23]. For instance, the *CBX7* expression resulted in a poor prognosis in ovarian clear cell adenocarcinoma [22]. Similarly, the *CDK1* overexpression was associated with a poor prognosis in colorectal cancer [23]. In addition, several studies reported that some CRs were associated with LC such as *CDK1* and *MGMT* [24, 25]. Despite some CRs advances, little is known about the roles of CRs in LUAD biology and a better understanding of CRs associated with the prognosis urgently requires identification.

In this study, we first constructed and evaluated two molecular prognostic subtypes (MPSs) based on the prognostic CRs. Subsequently, we established a six-gene prognostic signature based on two MPSs. Furthermore, we investigated the clinical and molecular features between differing MPSs and between differing risk subgroups, especially exploring the relationships of MPS and risk signature with TICs. This study will be helpful for better understanding the prognostic and immunological role of CRs in LUAD.

## RESULTS

**Figure 1:**
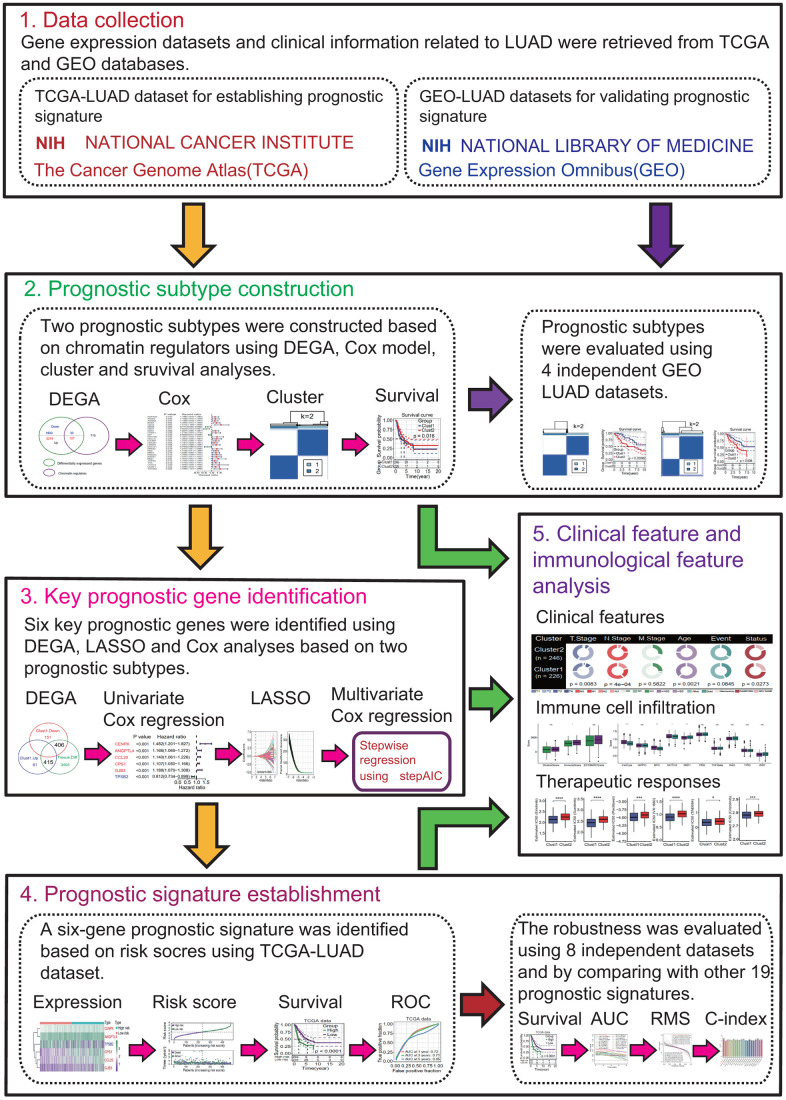
**Flow chart of the present study.** This study was divided into 5 research modules sequentially. (1) Data collection. Lung adenocarcinoma (LUAD) gene expression datasets were retrieved from the public gene expression omnibus (GEO) and the cancer genome atlas (TCGA) databases, respectively. (2) Prognostic subtype construction. Prognostic subtypes were constructed based on prognostic chromatin regulators. (3) Key prognostic gene identification. Key prognostic genes were identified by Cox regression and the least absolute shrinkage and selection operator (LASSO) analyses. (4) Prognostic signature establishment. Prognostic signature was established and evaluated. (5) Clinical feature and immunological feature analyses. The relationships of prognostic subtypes, prognostic genes and signature with clinical characteristics and immunological features were analyzed.

This study was divided into 5 research modules sequentially and the flow chart was presented in Figure 1: (1) Data collection. LUAD-related gene expression datasets and clinical data were retrieved from the public databases The Cancer Genome Atlas (TCGA) and Gene Expression Omnibus (GEO). The TCGA-LUAD dataset was used to construct prognostic subtypes and prognostic gene signature. The GEO-LUAD datasets were used to evaluate the validity of prognostic subtype and the robustness of prognostic gene signature. (2) Prognostic subtype construction. Two MPSs were constructed based on 27 differentially expressed chromatin regulators (DECRs) with significant prognostic value using the TCGA-LUAD dataset and the validity of MPSs was evaluated using 4 independent GEO-LUAD datasets. (3) Key prognostic gene identification. Six differentially expressed genes (DEGs) between two MPSs were identified to be key prognostic DEGs based on the univariate Cox regression analysis (UCRA), multivariate Cox regression analysis (MCRA) and least absolute shrinkage and selection operator (LASSO) analysis. (4) Prognostic signature establishment. A six-gene prognostic signature was established using the TCGA-LUAD dataset and its robustness was assessed using 8 independent GEO-LUAD datasets. Subsequently, the predictive ability of six-gene prognostic signature was evaluated by comparing with 19 reported prognostic signatures. (5) Clinical and immunological features analyses. The associations of MPS, key prognostic genes and risk score with clinical and immunological features were investigated. Potential responses of LUAD patients to immunotherapy and chemotherapy were predicted in different MPSs and in different risk subgroups, respectively.

### Twenty-seven DECRs were correlated with the prognosis in LUAD

**Figure 2:**
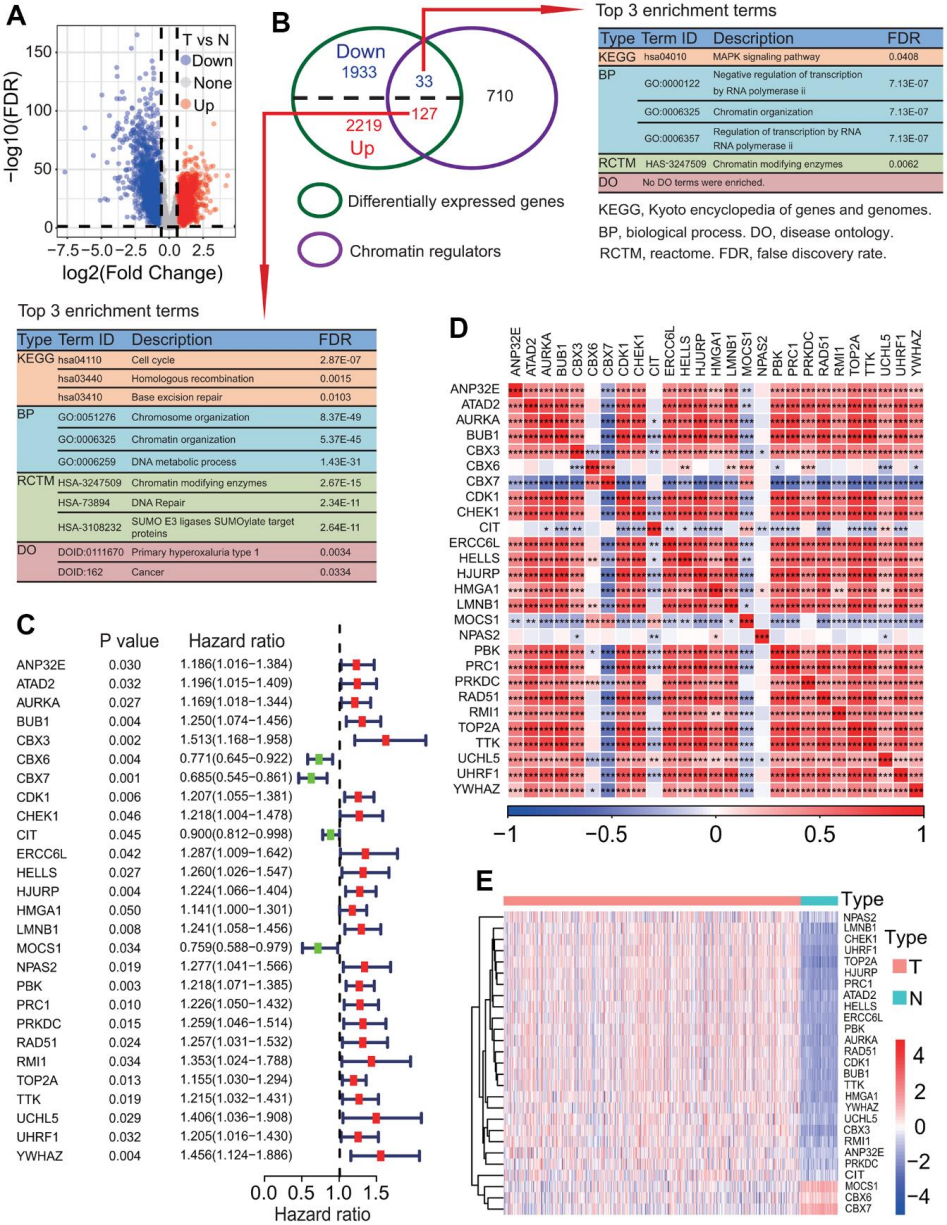
**Identification of key differentially expressed chromatin regulators associated with survival.** (**A**) Distribution of differentially expressed genes (DEGs) between lung adenocarcinoma (LUAD) and normal lung tissues. Totals of 2346 upregulated and 1966 downregulated DEGs were identified in LUAD tissue. (**B**) Identification of differentially expressed chromatin regulators (DECRs). Totals of 127 upregulated and 33 downregulated DECRs were identified in LUAD tissue. (**C**) Identification of key DECRs associated with overall survival (OS) rate of patients with LUAD. Univariate Cox regression analysis showed that 27 DECRs were associated with the OS of patients with LUAD. (**D**) Correlations between key prognostic DECRs in expression. Except three DECRs including *MOCS1*, *CBX7* and *CIT*, the expressions of the other 24 DECRs showed strong positive correlations on the whole. (**E**) Heat map of key prognostic DECRs in expression. Except three DECRs including *MOCS1*, *CBX7* and *CBX6*, the expression of the other 24 DECRs were upregulated in LUAD tissue.

To identify key prognostic CRs, a DEGA and a UCRA were performed. The DEGA result showed that totals of 2346 upregulated and 1966 downregulated genes were identified in LUAD tissue compared to normal lung tissue (Figure 2A and Supplementary Table 1). Among 127 upregulated and 33 downregulated genes were DECRs (Figure 2B and Supplementary Table 2). These DECRs, especially 127 upregulated DECRs, mainly involved in chromatin organization (GO:0051276, *p* = 8.37e-49), cell cycle (hsa04110, *p* = 2.87e-07), chromatin modifying enzymes (HAS-3247509, *p* = 2.67e-15), etc. (Figure 2B). The disease ontology (DO) result showed that 127 upregulated DECRs were mainly associated with cancer (DOID:162, *p* = 0.0334) (Figure 2B). The UCRA result showed that 27 of 160 DECRs had significant prognostic values (*p* < 0.05, Figure 2C). The 27 prognostic DECRs include 16 histone modifiers, 4 chromatin remodelers, 2 DNA methylators and 5 unknown types, respectively (Table 1). Except for *CBX6*, *CBX7*, *CIT* and *MOCS1*, the other 23 DECRs were risk factors (Figure 2C) and their expressions showed strong positive correlations with each other (most *p* < 0.001, Figure 2D). Except for *CBX7*, *CBX6* and *MOCS1*, the other 24 DECRs were significantly upregulated in LUAD tissues (adjusted *p* < 0.05, Figure 2E).

**Table 1:** Basic functional information of 27 prognostic chromatin regulators.

**Symbol**	**Type**	**Histone type**	**Methytor type**	**Function**
AURKA	Histone Modifier	Writer	/	Histone modification write (Histone phosphorylation)
BUB1	Histone Modifier	Writer	/	Histone modification write (Histone phosphorylation)
PBK	Histone Modifier	Writer	/	Histone modification write (Histone phosphorylation)
PRKDC	Histone Modifier	Writer	/	Histone modification write (Histone phosphorylation)
TTK	Histone Modifier	Writer	/	Histone modification write cofactor (Histone phosphorylation)
CDK1	Histone Modifier	Writer	/	Histone modification write (Histone phosphorylation)
CHEK1	Histone Modifier	Writer	/	Histone modification write (Histone phosphorylation)
CIT	Histone Modifier	Writer	/	Histone modification write cofactor (Histone phosphorylation)
ANP32E	Histone Modifier	Reader	/	Histone chaperone, Histone modification read
CBX3	Histone Modifier	Reader	/	Histone modification read
CBX6	Histone Modifier	Reader	/	Histone modification read
CBX7	Histone Modifier	Reader	/	Histone modification read
YWHAZ	Histone Modifier	Reader	/	Histone modification read
RAD51	Histone Modifier	Eraser	/	Histone modification erase (Histone ubiquitination)
UCHL5	Histone Modifier	Eraser	/	Histone modification erase cofactor (Histone ubiquitination)
HJURP	Histone Modifier	/	/	Histone chaperone
ATAD2	Chromatin Remodeler	/	/	Chromatin remodelling
HELLS	Chromatin Remodeler	/	/	Chromatin remodelling
NPAS2	Chromatin Remodeler	/	/	Chromatin remodelling
TOP2A	Chromatin Remodeler	/	/	Chromatin remodelling
RMI1	DNA Methylator	/	/	DNA modification
UHRF1	DNA Methylator Histone Modifier	Reader, Writer	Reader	Histone modification read, Histone modification write cofactor, DNA modification
LMNB1	/	/	/	Global heterochromatic changes
ERCC6L	/	/	/	/
HMGA1	/	/	/	/
MOCS1	/	/	/	/
PRC1	/	/	/	/

**Figure 3:**
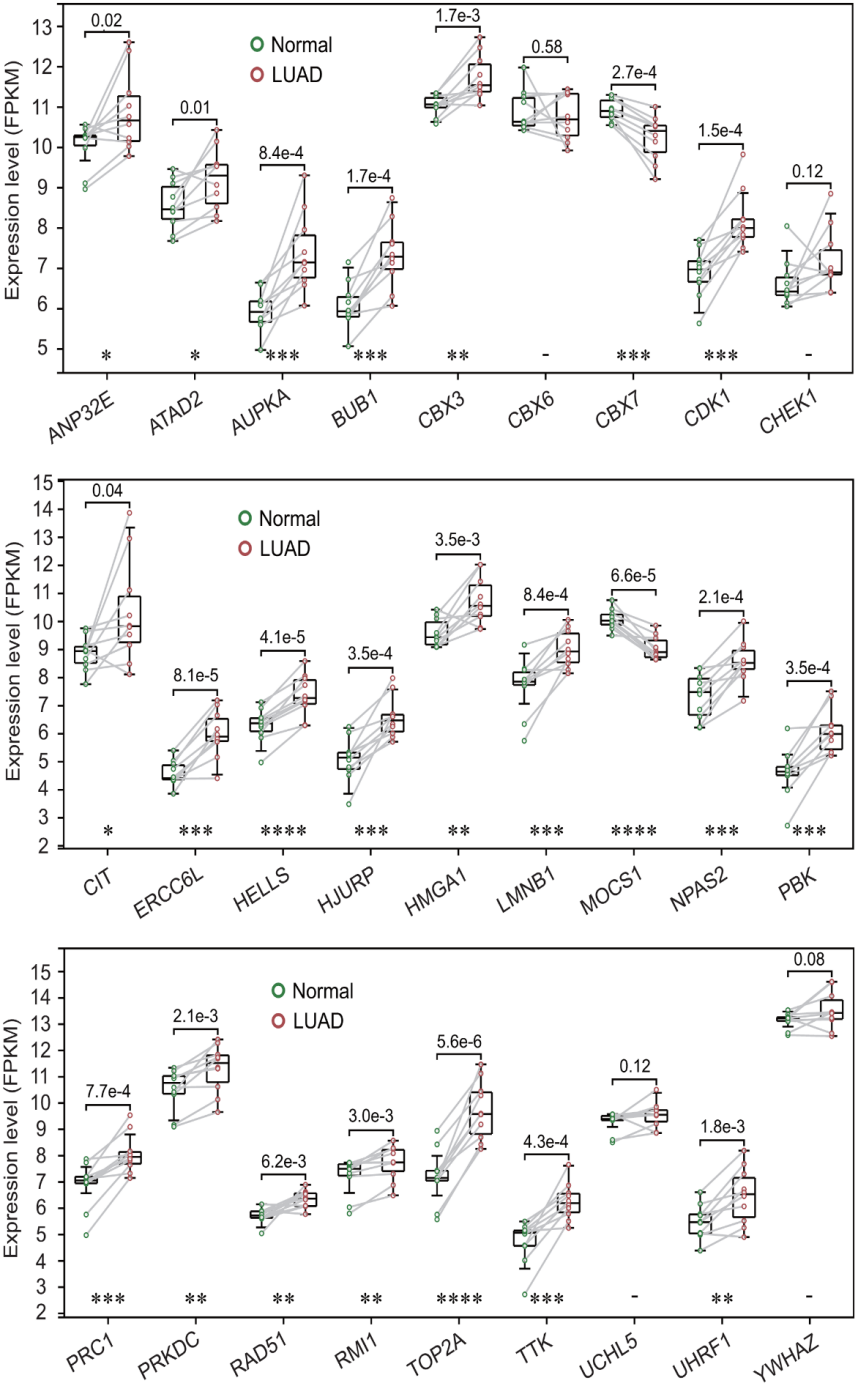
**Expression levels of 27 prognostic chromatin regulators between lung adenocarcinoma (LUAD) and normal lung tissues.** The expression levels of 27 prognostic chromatin regulators were compared between LUAD and normal lung tissues by a paired t-test method using 10 pairs of samples from 10 LUAD patients. The result was highly consistent with the TCGA-LUAD result.

To further investigate the expression levels of 27 prognostic DECRs between LUAD and normal lung tissues, the gene expressions of these 27 DECRs were compared between LUAD and normal lung tissues from 10 LUAD patients using a paired t-test method. The results showed that the expression levels of these 27 DECRs were highly consistent with the TCGA-LUAD results (Figure 3A and Supplementary Table 3).

### Two MPSs constructed by 27 prognostic DECRs were valid

**Figure 4:**
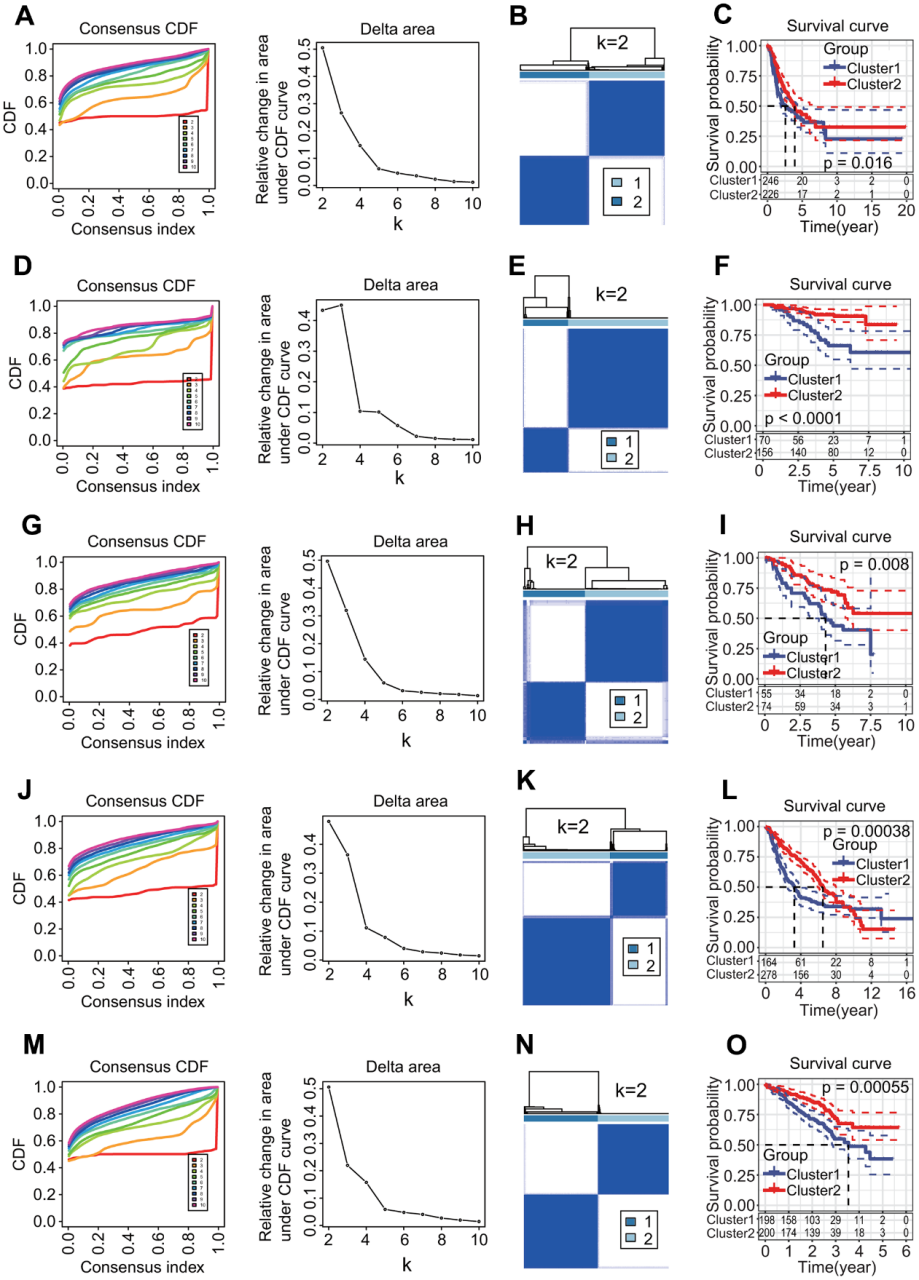
**Molecular prognostic subtypes construction based on 27 key prognostic differentially expressed chromatin regulators (DECRs) and the associations with overall survival (OS) of patients in lung adenocarcinoma (LUAD).** (**A**–**C**) TCGA-LUAD. (**D**–**F**) GSE31210. (**G**–**I**) GSE50081. (**J**–**L**) GSE68465. (**M**–**O**) GSE72094. (**A**, **D**, **G**, **J**, **M**) Cumulative distribution function (CDF) curve and CDF delta area curve. Clustering stability increasing curves were plotted from k = 2 to 9 for five gene expression datasets including TCGA-LUAD, GSE31210, GSE50081, GSE68465 and GSE72094. (**B**, **E**, **H**, **K**, **N**) Consensus clustering heat map. The k = 2 was selected to cluster samples for each of five gene expression datasets, and LUAD patients were divided into two clusters. (**C**, **F**, **I**, **L**, **O**) Survival curve. Two clusters were significantly associated with the OS of LUAD patients in five independent datasets (*p* = 0.016, < 0.0001, = 0.008, = 0.00038, = 0.00055, respectively).

To investigate the relationships of 27 prognostic DECRs with LUAD prognostic subtype, a consensus clustering for the TCGA-LUAD samples was performed based on the gene expression profiles of 27 prognostic DECRs. According to clustering stabilities increasing from k = 2 to 9 (Figure 4A), k = 2 was selected for sample cluster and LUAD samples were clustered into two clusters called Cluster1 (C1 subtype) and Cluster2 (C2 subtype) (Figure 4B). The LUAD patients in the C2 subtype had a higher OS rate than those in the C1 subtype (*p* = 0.016, Figure 4C).

To evaluate the validity of two MPSs, four independent GEO-LUAD gene expression datasets including GSE31210, GSE50081, GSE68465 and GSE72094 were clustered using the same clustering method, respectively. The k = 2 was the optimal parameter for each of four datasets (Figure 4D, 4G, 4J, 4M) and LUAD samples were clearly divided into two clusters (Figure 4E, 4H, 4K, 4N). There was a significant difference in the OS rate between two clusters (*p* < 0.0001, = 0.008, = 0.00038, = 0.00055, respectively, Figure 4F, 4I, 4L, 4O).

**Figure 5:**
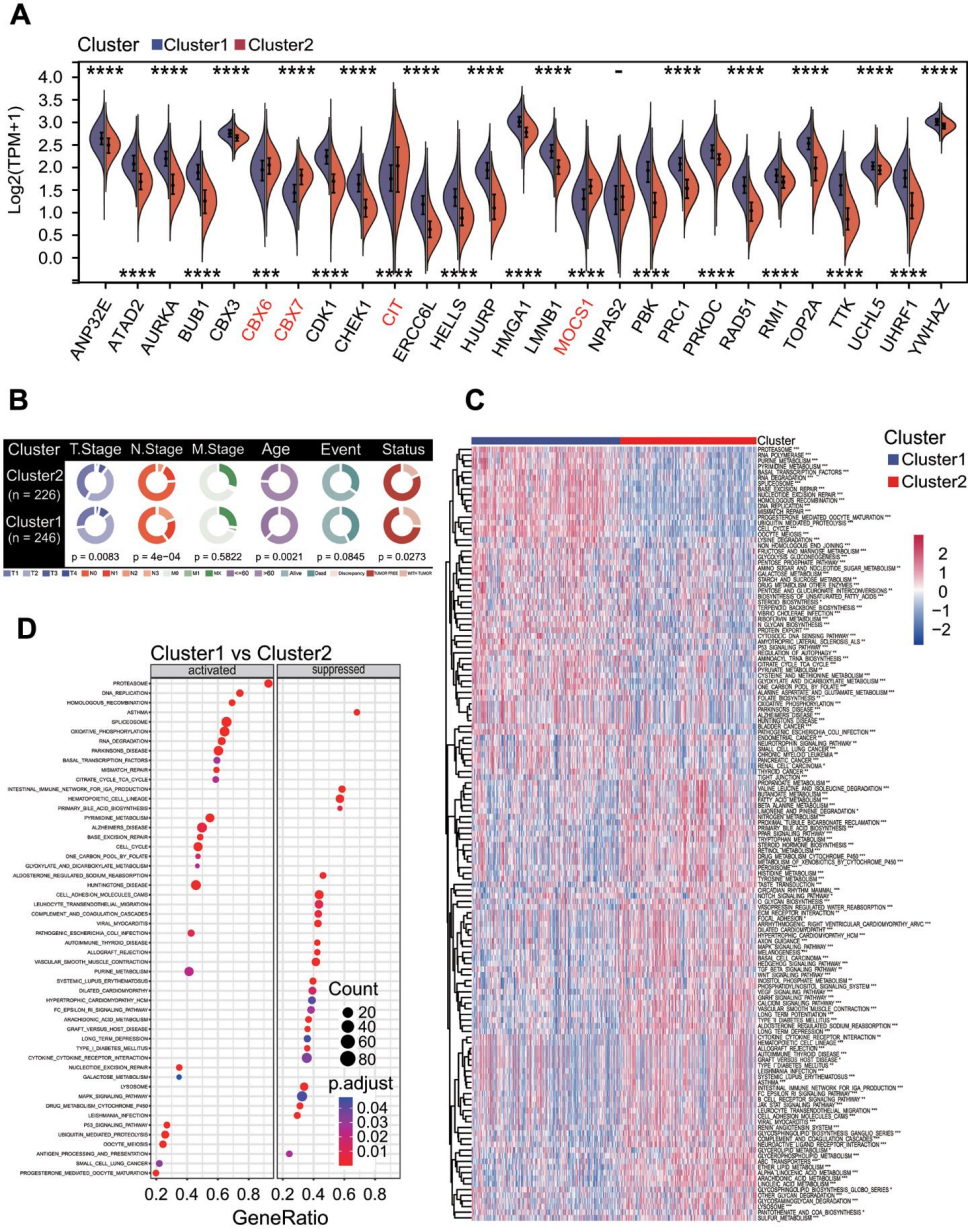
**Correlations of molecular prognostic subtype with clinical features and KEGG pathway enrichment.** (**A**) Expression of 27 prognostic chromatin regulators between two clusters. (**B**) Comparison of clinical features between two clusters. Chi-square test showed the significant differences in T staging (*p* = 0.0083), N staging (*p* = 4e-04), age (*p* = 0.0021) and cancer status (*p* = 0.0273). (**C**) Gene set variation analysis. Totals of 184 KEGG pathways were significantly enriched between two clusters. (**D**) Gene set enrichment analysis. The top 26 significantly enriched KEGG pathways were shown between two clusters.

The expression analysis showed that 26 of 27 prognostic DECRs had significant differences between two clusters except for *NPAS2* (Figure 5A), and 4 DECRs including *CBX6*, *CBX7*, *MOCS1* and *CIT* were highly expressed in the C2 subtype*.* Among the 26 DECRs, except for *CIT*, the expression levels of 25 DECRs between the C1 subtype and LUAD group showed the same trends, and those between the C2 subtype and normal lung tissue, respectively (Figures 2E, 5A).

### MPSs were correlated with clinical and biological features

To explore the correlations of MPSs with clinical features, clinical features between C1 and C2 subtypes were compared. The results showed that there were significant differences in the T staging (chi-square *p* = 0.0083), N staging (chi-square *p* = 4e-04), age (chi-square *p* = 0.0021) and cancer status (chi-square *p* = 0.0273) between these two MPSs (Figure 5B). The LUAD patients in the C2 subtype had a lower T staging and N staging than those in the C1 subtype.

To investigate the correlations of MPSs with biological features, functional enrichment analyses were performed to discover potential functional differences between these two MPSs by using GSVA and GSEA methods. The GSVA result showed that a total of 184 KEGG pathways was significantly enriched (*p* < 0.05, Figure 5C). Among these KEGG pathways, some key oncogenic signaling pathways (OSPs) in the C2 subtype were lower active than those in the C1 subtype, such as cell cycle and P53 signaling pathway (both *p* < 0.001, Figure 5C). The GSEA result was consistent with that obtained by the GSVA method (Figure 5D) and some OSPs including cell cycle (*p* = 4.60e-09) and P53 signaling pathway (*p* = 2.12e-04) were significantly downregulated in the C2 subtype (Figure 5D).

**Figure 6:**
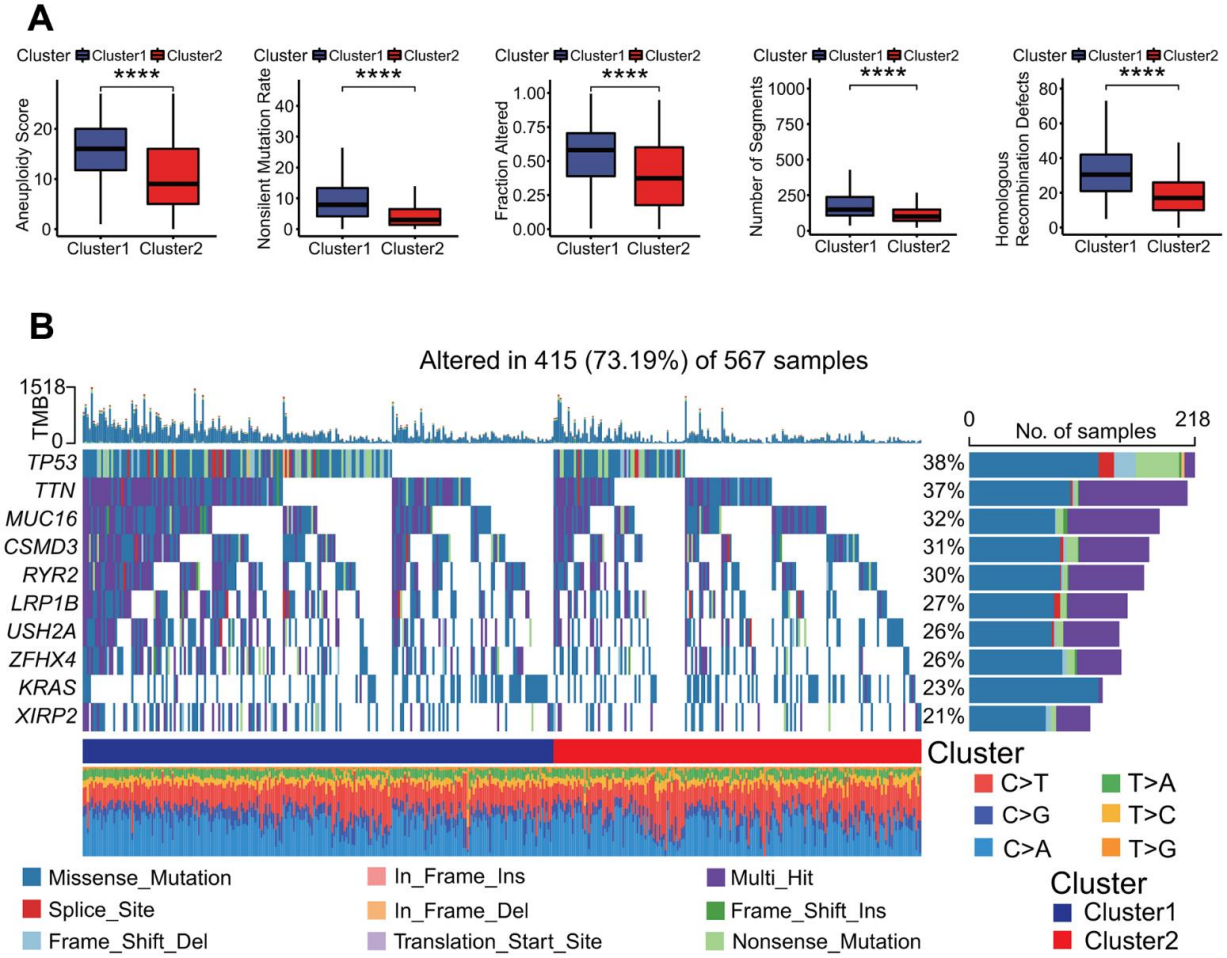
**Genomes alterations between two clusters.** (**A**) Molecular features of genome alterations. There was higher aneuploidy score, nonsilent mutation rate, fraction altered of genome, number of segments and homologous recombination defects in the cluster 1. *p < 0.05, **p < 0.01, ***p < 0.001 and ****p < 0.0001. (**B**) Gene mutation profiles between two clusters. The mutation frequencies of top 10 genes including *TP53*, *TTN* and *MUC16* and so on had significant differences in two clusters, and there was higher mutation frequency in the cluster 1.

To further investigate the characteristics of genomic variations between two MPSs, a somatic mutation analysis was performed. The result showed that the aneuploidy score, non-silent mutation rate, fraction altered, number of segments and homologous recombination defects in the C2 subtype were significantly lower than those in the C1 subtype (Wilcoxon test all *p* < 0.0001, Figure 6A). The mutation rate and main mutation type of top 10 altered genes were significantly different between two MPSs (Figure 6B).

### MPSs were correlated with immunological feature and therapeutic response

**Figure 7:**
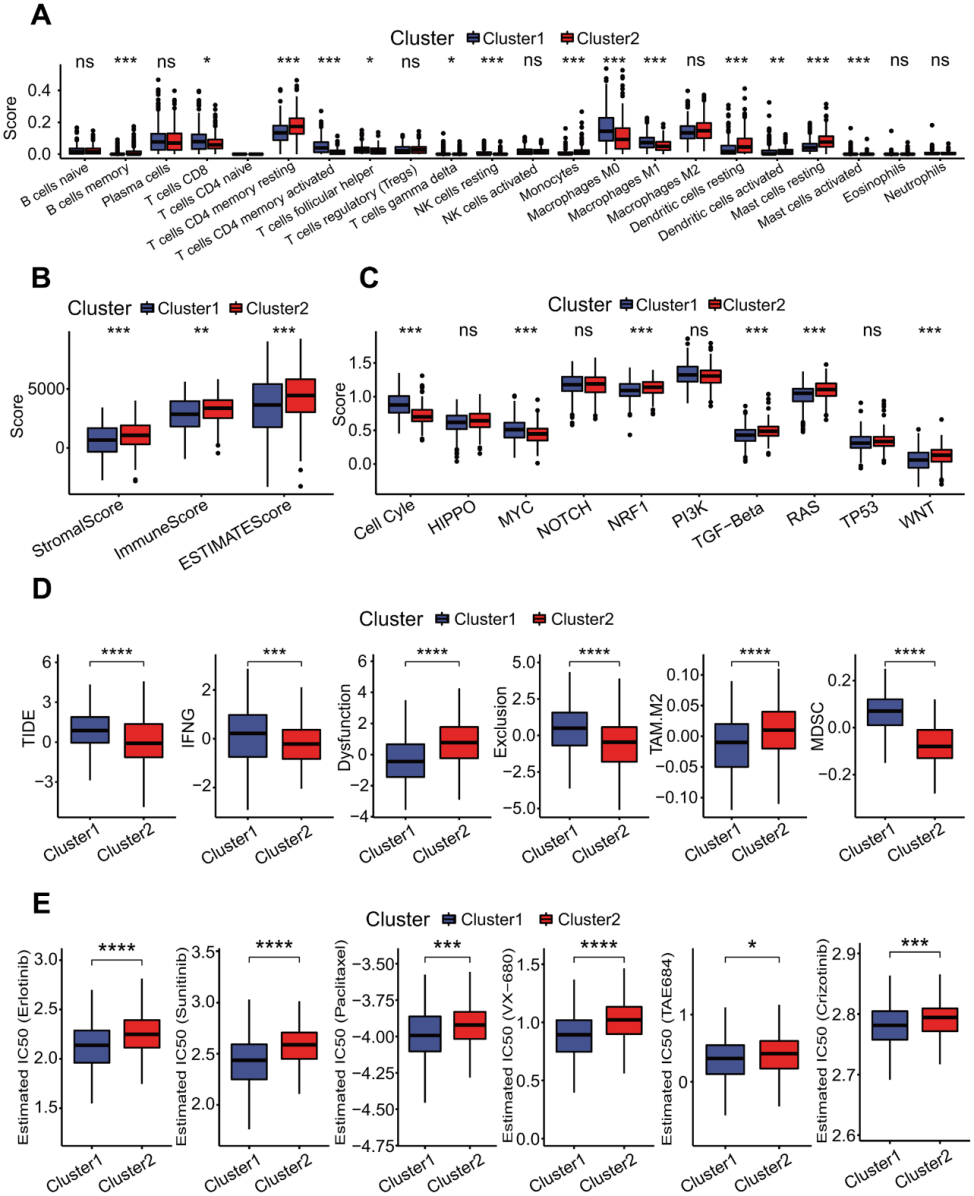
**Correlations of molecular prognostic subtypes with immunological features.** (**A**) Infiltration comparisons of 22 immune cell types. The scores of some immune cell types had significant differences between two clusters, such as T cells CD4 memory resting, mast cells resting and dendritic cells resting with higher scores in the cluster 2. *p < 0.05, **p < 0.01 and ***p < 0.001. (**B**) Infiltration comparison of stromal and immune cells. The cluster 2 had higher stromal score, immune score and ESTIMATE score. *p < 0.05, **p < 0.01 and ***p < 0.001. (**C**) Comparisons of activities of 10 oncogenic signaling pathways. The scores of cell cycle, MYC, NRF1, TGF-beta, RAS and WNT pathways had significant differences between two clusters, and these oncogenic signaling pathways had lower activities in the cluster 2. *p < 0.05, **p < 0.01, ***p < 0.001. (**D**) Comparisons of tumor immune dysfunction and exclusion (TIDE) score, interferon-gamma (IFNG), T cell dysfunction (Dysfunction), T cell exclusion (Exclusion), tumor-associated macrophages M2 (TAM.M2) and myeloid-derived suppressor cells (MDSC). These terms had significant differences between two clusters, and TIDE, IFNG, Exclusion and MDSC in the cluster 1 were significantly higher than those in the cluster 2. Dysfunction and TAM. M2 in the cluster 1 were significantly lower than those in the cluster 2. *p < 0.05, **p < 0.01, ***p < 0.001 and ****p < 0.0001. (**E**) Comparisons of estimated IC50 values for 6 conventional chemotherapy agents. The estimated IC50 values for erlotinib, sunitinib, paclitaxel, VX-680, TAE684 and crizotinib in the cluster 1 were significantly lower than those in the cluster 2. *p < 0.05, **p < 0.01, ***p < 0.001 and ****p < 0.0001.

To elucidate the relationships of MPSs with immunological features, a comprehensive immunological analysis was implemented. A CIBERSORT-based analysis showed that the relative abundances of 14 of 22 immune cell types had significant differences between two MPSs (Wilcoxon test *p* < 0.05 or < 0.01 or < 0.001, Figure 7A). Among these 14 immune cells types, the abundances of 6 immune cell types including B cells memory (*p* < 0.001), T cells CD4 memory resting (*p* < 0.001), monocytes (*p* < 0.001), dendritic cells resting (*p* < 0.001), dendritic cells activated (*p* < 0.01) and mast cells resting (*p* < 0.001) were significantly higher in the C2 subtype than those in the C1 subtype (Figure 7A). Inversely, the abundances of 8 immune cell types including T cells CD8 (*p* < 0.05), T cells CD4 memory activated (*p* < 0.001), T cells follicular helper (*p* < 0.05), T cells gamma delta (*p* < 0.05), NK cells resting (*p* < 0.001), macrophages M0 (*p* < 0.001), macrophages M1 (*p* < 0.001) and mast cells activated (*p* < 0.001) were significantly lower in the C2 subtype (Figure 7A). The stromal score (*p* < 0.001), immune score (*p* < 0.01) and ESTIMAT score (*p* < 0.001) in the C2 subtype were significantly higher than those in the C1 subtype (Figure 7B). Two OSPs including cell cycle (*p* < 0.001) and MYC (*p* < 0.001) in the C2 subtype were less active than those in the C1 subtype (Figure 7C), and 4 OSPs containing NRF1 (*p* < 0.001), TGF-beta (*p* < 0.001), RAS (*p* < 0.001) and WNT (*p* < 0.001) in the C2 subtype were more active than those in the C1 subtype (Figure 7C). The TIDE result showed that the TIDE score (*p* < 0.001), IFNG score (*p* < 0.01), exclusion score (*p* < 0.001) and MDSC score (*p* < 0.001) were significantly lower in the C2 subtype, and the dysfunction score (*p* < 0.001) and TAM.M2 score (*p* < 0.001) were significantly higher in the C2 subtype (Figure 7D). The estimated IC50 values for 6 conventional chemotherapy agents including erlotinib (*p* < 0.0001), sunitinib (*p* < 0.0001), paclitaxel (*p* < 0.001), VX-680 (*p* < 0.0001), TAE684 (*p* < 0.05) and crizotinib (*p* < 0.001) in the C2 subtype were significantly higher than those in the C1 subtype (Figure 7E).

### Six key DEGs between two MPSs were correlated with the prognosis in LUAD

**Figure 8:**
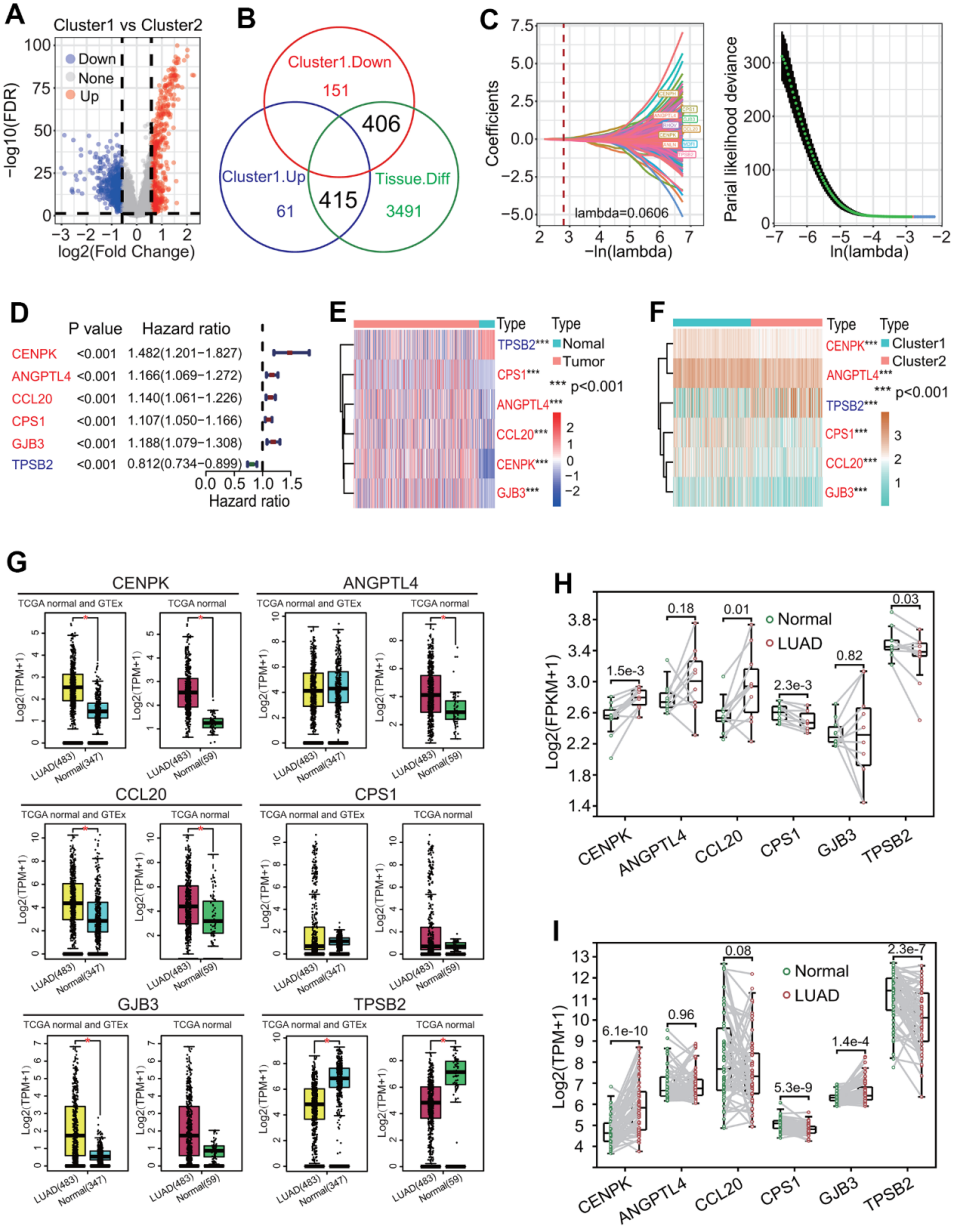
**Identification of key differentially expressed genes (DEGs) between two clusters.** (**A**) Distribution of DEGs between two clusters. Totals of 466 upregulated and 557 downregulated DEGs were identified in the cluster 1. (**B**) Identification of key DEGs between two clusters. An overlap analysis showed that totals of 821 dysregulated DEGs were important between two clusters. (**C**) LASSO Cox analysis. A 10-round cross validation was performed to prevent overfitting and 10 DEGs (*CENPH*, *RHOV*, *ANLN*, *MDFI*, *TPSB2*, *CPS1*, *ANGPTL4*, *CCL20*, *CENPK*, *GJB3*) had the most important feature when lambda was equal to 0.0606. (**D**) Univariate Cox regression analysis. Six DEGs including *TPSB2*, *CPS1*, *ANGPTL4*, *CCL20*, *CENPK* and *GJB3* had significant prognostic values (all *p* < 0.001). (**E**) Heat map of expressions of six DEGs between lung adenocarcinoma (LUAD) and normal lung tissues. Five DEGs including *CPS1*, *ANGPTL4*, *CCL20*, *CENPK* and *GJB3* were upregulated expressed and *TPSB2* was downregulated expressed in LUAD tissue. (**F**) Heat map of expressions of six DEGs between two clusters. Five DEGs including *CPS1*, *ANGPTL4*, *CCL20*, *CENPK* and *GJB3* were upregulated expressed and *TPSB2* was downregulated expressed in the cluster 1. (**G**–**I**) The expression levels of 6 prognostic genes between LUAD and normal lung tissues based on GEPIA (gene expression profiling interactive analysis), real RNA-seq and GSE19804 datasets.

**Figure 9:**
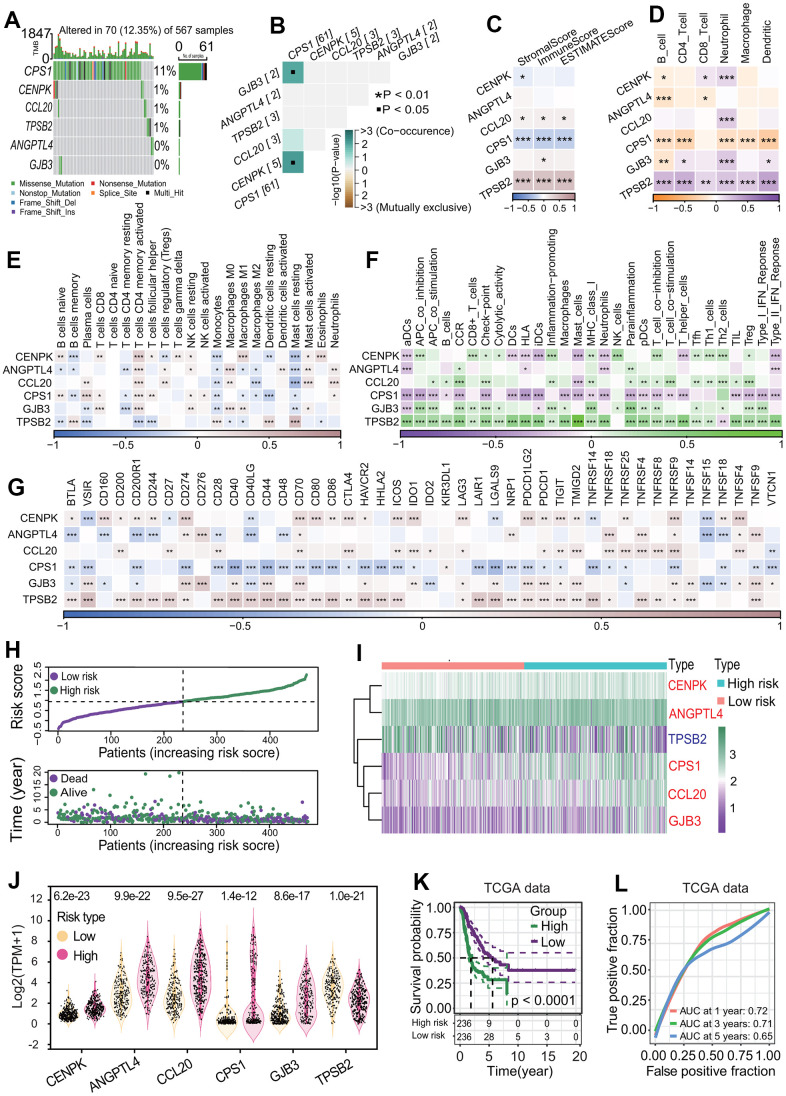
**Correlation of six differentially expressed genes (DEGs) with immunity and establishment of six-gene prognostic signature.** (**A**) Gene mutation profiles of six DEGs. Only 12.35% of LUAD samples had one or more mutations in six DEGs (*CPS1*, *ANGPTL4*, *CCL20*, *CENPK*, *GJB3*, *TPSB2*). (**B**) Co-occurrence and mutually exclusive of gene mutation. Six DEGs had lower co-occurrence and are mutually exclusive. (**C**) Correlation of six DEGs with stromal and immune cell infiltration. Two genes including *CPS1* and *TPSB2* were strongly negatively and positively correlated with three infiltration scores of two cells including stromal and immune cells, respectively. *p < 0.05, **p < 0.01 and ***p < 0.001. (**D**) Correlations of six DEGs with infiltrations of six immune cells. The expressions of six DEGs had significant correlations with one or more types of immune cells. In particular, *CPS1* and *TPSB2* were strongly negatively and positively correlated with immune infiltrations of six types of immune cells, respectively. *p < 0.05, **p < 0.01 and ***p < 0.001. (**E**) Correlations of six DEGs with 22 immune cell types. In general, the expressions of 6 DEGs (*CPS1*, *ANGPTL4*, *CCL20*, *CENPK*, *GJB3*, *TPSB2*) were strongly correlated with 22 immune cell types. *p < 0.05, **p < 0.01 and ***p < 0.001. (**F**) Correlations of six DEGs with 29 immune cell types. The expressions of six genes had very strong correlations with multiple immune cell types. In particular, *CPS1* and *TPSB2* were strongly negatively and positively correlated with immune infiltrations of 29 types of immune cells, respectively. *p < 0.05, **p < 0.01 and ***p < 0.001. (**G**) Correlations of six DEGs with 44 immune checkpoint genes. The expressions of six DEGs had very strong correlations with the expressions of multiple checkpoint genes. Especially, *CPS1* and *TPSB2* were strongly negatively and positively correlated with most checkpoint genes, respectively. *p < 0.05, **p < 0.01 and ***p < 0.001. (**H**) Risk score distribution and survival overview of LUAD patients. According to the median risk score, LUAD patients were divided into high- and low-risk subgroups. (**I**, **J**) Expression levels of six prognostic genes between two risk subgroups. Five genes including *CENPK*, *ANGPTL4*, *CPS1*, *CCL20* and *GJB3* were highly expressed and *TPSB2* gene were lowly expressed in the high-risk subgroup. (**K**) Survival curve. LUAD patients in the low-risk subgroup had a higher OS rate than that in the high-risk subgroup (*p* < 0.0001). (**L**) Receiver operating characteristic (ROC) curve. The areas under the curve (AUCs) associated with 1-year, 3-year and 5-year survival were 0.72, 0.71 and 0.65, respectively.

To identify key DEGs associated with the survival of LUAD patients between two MPSs, a comprehensive analysis including DEGA, UCRA, LASSO and MCRA was performed. The DEGA result showed that totals of 476 upregulated and 557 downregulated genes were identified in the C1 subtype compared with the C2 subtype (Figure 8A and Supplementary Table 4). Compared with the DEGs obtained from LUAD and normal lung tissues, a total of 821 dysregulated DEGs was common (Figure 8B and Supplementary Table 5). The UCRA showed that 258 of 821 DEGs had significant prognostic values (Supplementary Table 6). The LASSO analysis showed that 10 (*CENPH*, *RHOV*, *ANLN*, *MDFI*, *TPSB2*, *CPS1*, *ANGPTL4*, *CCL20*, *CENPK*, *GJB3*) of 258 DEGs with UCRA prognostic value had the most important feature when lambda was equal to 0.0606 (Figure 8C). The MCRA by AIC criterion showed that 6 (*TPSB2*, *CPS1*, *ANGPTL4*, *CCL20*, *CENPK*, *GJB3*) of 10 DEGs with the most important feature had the greatest fitting degree. Except for *TPSB2*, the expressions of the other five prognostic DEGs (*CPS1*, *ANGPTL4*, *CCL20*, *CENPK*, *GJB3*) were significantly negatively correlated with the survival of LUAD patients (all *p* < 0.001, Figure 8D) and their expressions were significantly upregulated in LUAD tissues (all *p* < 0.001, Figure 8E) and in the C1 subtype (all *p* < 0.001, Figure 8F). The expression analysis based on the GEPIA (gene expression profiling interactive analysis), real transcriptome data and GSE19804 datasets showed that the expression levels of 6 prognostic DEGs were consistent with those from the TCGA dataset between LUAD and normal lung tissues (Figure 8G–8I and Supplementary Table 3). Except for *CPS1*, each of the other five prognostic DEGs had a low mutation rate (Figure 9A) and a lower co-occurrence and mutually exclusive (Figure 9B).

### Six key prognostic DEGs were correlated with immunological features

To investigate the relationships of six key prognostic DEGs with immunological features, a comprehensive immunological analysis was performed. Among six key prognostic DEGs, *CPS1* and *TPSB2* were negatively and positively correlated with the stromal score, immune score and ESTIMATE score, respectively (all *p* < 0.001, Figure 9C). Similarly, *CPS1* and *TPSB2* were negatively and positively correlated with five immune cell types including B cells, CD4 T cells, neutrophils, macrophages and dendritic cells (all *p* < 0.001, Figure 9D). Furthermore, a CIBERSORT-based analysis showed that there were generally stronger correlations of six key prognostic DEGs with 22 immune cell types, especially T cells CD4 memory activated, monocytes and mast cells resting (Figure 9E). An ssGSEA-based analysis showed that six key prognostic DEGs were strongly correlated with 29 immune cell types overall, especially *CPS1* and *TPSB2* (most *p* < 0.001, Figure 9F). As a whole, six key prognostic DEGs were strongly correlated with 44 immune checkpoints in expression (Figure 9G). In particular, *CPS1* and *TPSB2* were strongly negatively and positively correlated with these immune checkpoints in expression, respectively (most *p* < 0.001, Figure 9G).

### Six-gene prognostic signature is robust in predicting the prognosis in LUAD

To further explore the correlation of six key prognostic DEGs with the survival, a six-gene prognostic model was constructed using the TCGA-LUAD dataset. In the light of the MCRA method by AIC criterion, the regression coefficient of each of six DEGs was calculated and the risk score was formulated:


$$\begin{array}{l} \text{Risk score}=0.235*\text{Exp}(CENPK)\\ \;\;\;+0.104*\text{Exp}(ANGPTL4)+0.088*\text{Exp}(CCL20)\\ \;\;\;+0.061*\text{Exp}(CPS1)+0.138*\text{Exp}(GJB3)\\ \;\;\;-0.134*\text{Exp}(TPSB2). \end{array}$$

**Figure 10:**
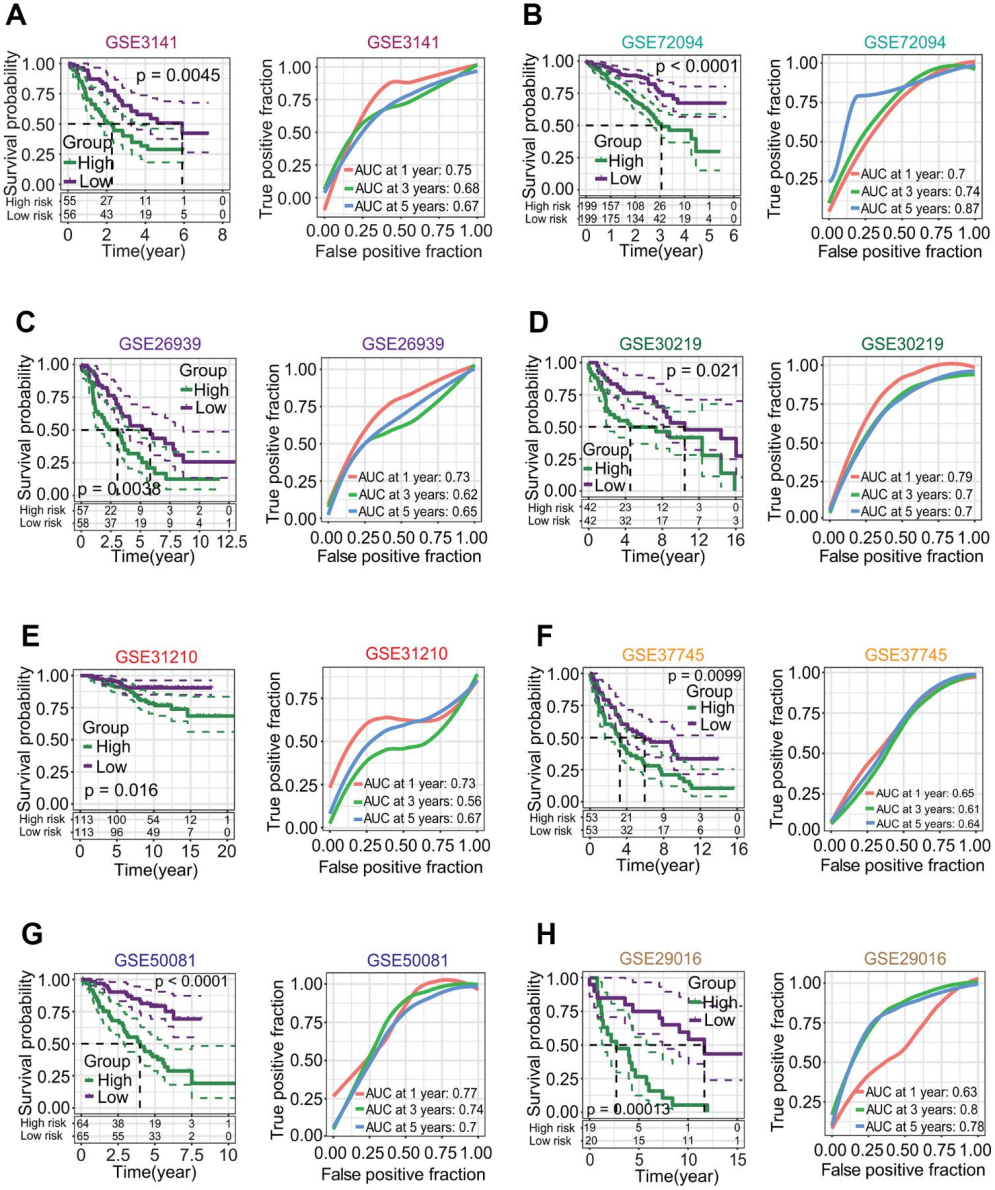
**Correlations of six-gene signature with overall survival (OS) of patients in lung adenocarcinoma (LUAD).** (**A**–**H**) Survival analysis based on eight independent LUAD datasets from the gene expression omnibus (GEO) database. Eight GEO-LUAD datasets were GSE3141, GSE72094, GSE26939, GSE30219, GSE31210, GSE37745, GSE50081 and GSE29016. For each dataset, LUAD patients in the low-risk subgroup had a higher OS rate than those in high-risk subgroup (*p* = 0.0045, < 0.0001, = 0.0038, = 0.021, = 0.016, = 0.0099, < 0.0001, = 0.00013, respectively), and the AUCs associated with 5-year survival were 0.67, 0.87, 0.65, 0.7, 0.67, 0.64, 0.7 and 0.78, respectively.

The risk score of each patient was calculated by the risk score formula. According to the median risk score, LUAD patients were divided into high-risk and low-risk subgroups (Figure 9H). Except for *TPSB2*, the other five prognostic DEGs including *CENPK*, *ANGPTL4*, *CPS1*, *CCL20* and *GJB3* were significantly upregulated in the high-risk subgroup (all *p* < 0.05, Figure 9I, 9J). The LUAD patients in the high-risk subgroup had a poorer OS rate than those in the low-risk subgroup (*p* < 0.0001, Figure 9K), and the AUCs of 1-year, 3-year and 5-year correlated with the survival were separately 0.72, 0.71 and 0.65 in the ROC curve (Figure 9L). Survival analysis based on eight independent GEO-LUAD datasets showed that the OS rate of LUAD patients in the low-risk subgroup was significantly higher than that in the high-risk subgroup for each GEO-LUAD dataset (GSE3141, *p* = 0.0045, Figure 10A; GSE72094, *p* < 0.0001, Figure 10B; GSE26939, *p* = 0.0038, Figure 10C; GSE30219, *p* = 0.021, Figure 10D; GSE31210, *p* = 0.016, Figure 10E; GSE37745, *p* = 0.0099, Figure 10F; GSE50081, *p* < 0.0001, Figure 10G; GSE29016, *p* = 0.00013, Figure 10H). The AUCs of 5-year associated with the survival were 0.67 (GSE3141, Figure 10A), 0.87 (GSE72094, Figure 10B), 0.65 (GSE26939, Figure 10C), 0.7 (GSE30219, Figure 10D), 0.67 (GSE31210, Figure 10E), 0.64 (GSE37745, Figure 10F), 0.7 (GSE50081, Figure 10G) and 0.78 (GSE29016, Figure 10H) for eight independent datasets, respectively.

**Figure 11:**
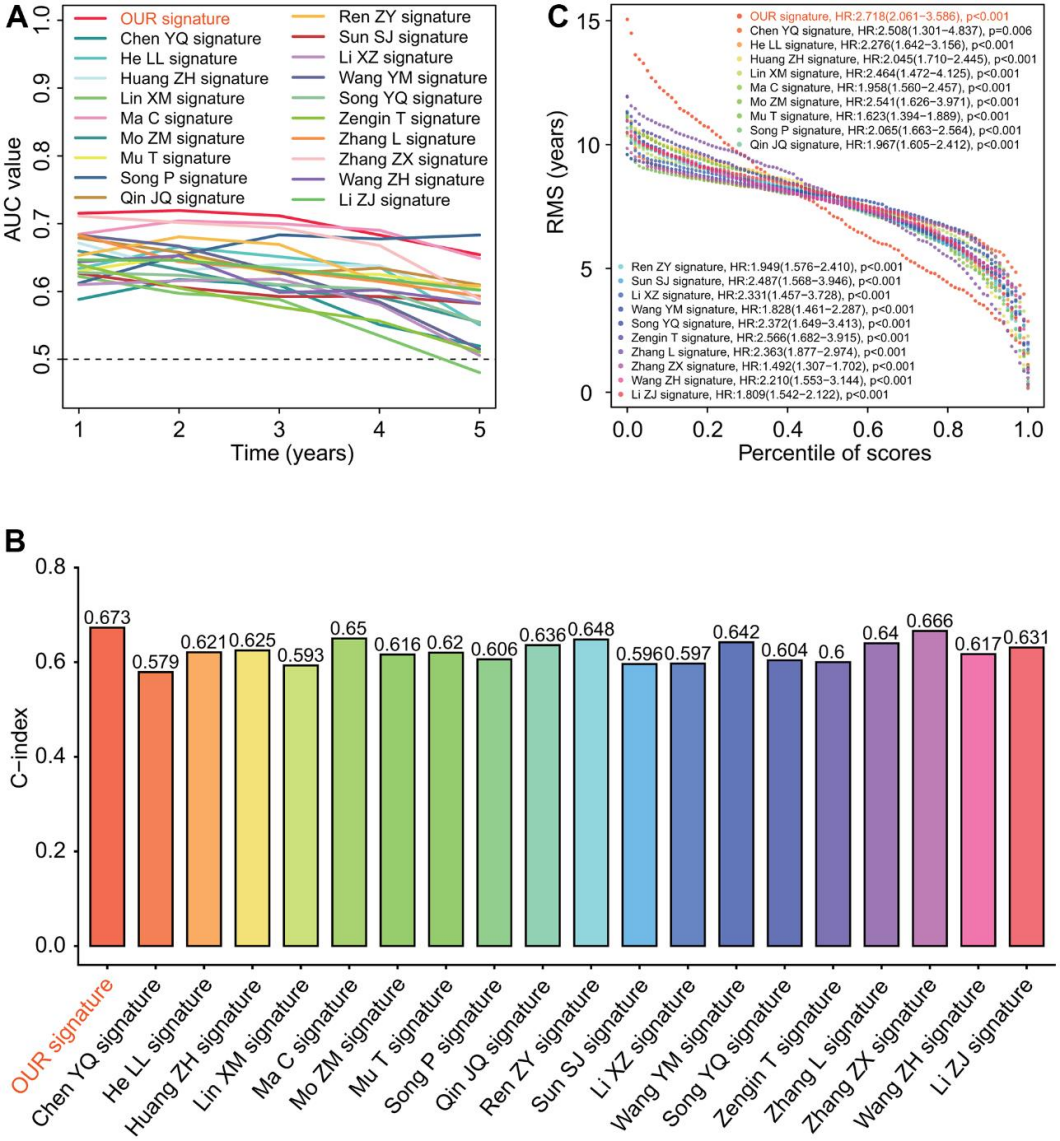
**Predictive performances of 20 prognostic signatures.** (**A**) AUC (area under the curve of receiver operating characteristic) change curve. Six-gene prognostic signature had the highest AUC values associated with 1-year and 3-year survivals and the next highest AUC value associated with 5-year survival. (**B**) Concordance index (C-index). Six-gene prognostic signature had the highest C-index 0.673 among 20 prognostic signatures. (**C**) Restricted mean survival (RMS) curve. Six-gene prognostic signature we developed had the highest hazard rate among 20 prognostic signatures.

To further evaluate the robustness of six-gene prognostic signature in predicting the prognosis, we compared the prognostic significance and predictive performance of six-gene prognostic signature with 19 reported prognostic signatures [26–45] (Supplementary Figures 1–3). The results showed that the six-gene prognostic signature in this study had the highest AUC values associated with 1-year and 3-year survival, and the next highest AUC value associated with 5-year survival (Figure 11A). Six-gene prognostic signature had the highest concordance index (C-index) 0.673 (Figure 11B) and hazard ratio (HR) 2.718 of restricted mean survival (RMS) time (Figure 11C).

### Risk score was correlated with clinical and biological features

**Figure 12:**
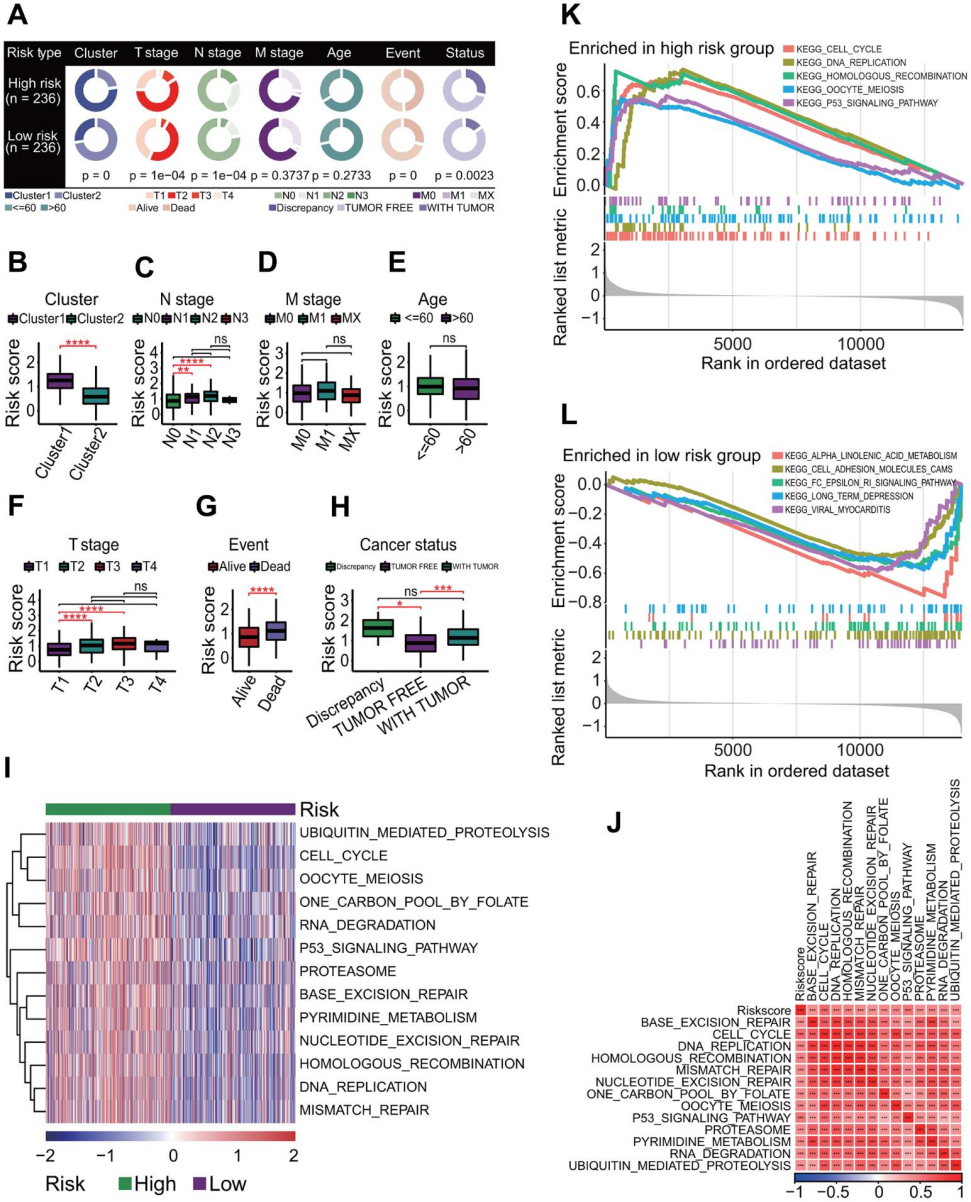
**Correlations of risk score with clinical characteristics and biological pathways.** (**A**) Correlations of risk score with clinical characteristics. Risk score was significantly correlated with prognostic cluster (*p* = 0), T staging (*p* = 1e-04), N staging (*p* = 1e-04), survival status (*p* = 0) and cancer status (*p* = 0.0023). (**B**) Comparisons of risk scores for patients between two clusters. Risk score in the cluster 1 was significantly higher than that in the cluster 2. ****p < 0.0001. (**C**) Comparisons of risk scores for patients between four N stages. Risk scores of patients with stage N0 were significantly lower than those with stage N1 and those with stage N2. **p < 0.01 and ***p < 0.001. (**D**) Comparisons of risk scores for patients between three M stages. The risk scores had no significant differences between three M stages. (**E**) Comparison of risk scores for patients between <= 60 and > 60 age subgroups. The risk scores had no significant differences between two age subgroups. (**F**) Comparisons of risk scores for patients between four T stages. Risk scores of patients with stage T1 were significantly lower than those with stage T2 and those with stage T3. ****p < 0.0001. (**G**) Comparison of risk scores for patients between two survival statuses. The risk scores for the living LUAD patients were significantly lower than those for dead patients. ****p < 0.0001. (**H**) Comparisons of risk scores for patients between three cancer statuses. The risk scores for patients with tumor free were significantly lower than those with discrepancy tumor. *p < 0.05 and ***p < 0.001. (**I**) Gene set variation analysis. At the correlation of risk score with KEGG pathway > 0.4 or < -0.4 and *p* < 0.001, thirteen KEGG pathways were positively correlated with risk score. (**J**) Correlations of risk score with KEGG pathways. There was a strong positive correlation between risk score and 13 KEGG pathways. ***p < 0.001. (**K**) Top 5 KEGG pathways enriched in the high-risk group. Five KEGG pathways were separately cell cycle, DNA replication, homologous recombination, oocyte meiosis and P53 signaling pathway. (**L**) Top 5 KEGG pathways enriched in the low-risk group. Five KEGG pathways were separately ALPHA linolenic acid metabolism, cell adhesion molecules (CAMs), Fc epsilon RI signaling pathway, long term depression and viral myocarditis.

To investigate the relationships of risk score with clinical and biological features, these features between the high- and low-risk subgroups were compared. The results showed that there were significant differences in the MPS (chi-square *p* = 0), T staging (chi-square *p* = 1e-04), N staging (chi-square *p* = 1e-04), survival status (chi-square *p* = 0) and cancer status (chi-square *p* = 0.0023) between the two risk subgroups (Figure 12A). The risk scores for LUAD patients in the C2 subtype were significantly lower than those in the C1 subtype (*p* < 0.0001, Figure 12B). The risk scores for LUAD patients with stage N0 were significantly lower than those for patients with stage N1 and patients with stage N2 (*p* < 0.001 and < 0.0001, respectively, Figure 12C). Similarly, the risk scores for LUAD patients with stage T1 were significantly lower than those for patients with stage T2 and patients with stage T3 (both *p* < 0.0001, Figure 12F). The risk scores for LUAD patients in the living subgroup were significantly lower than those in the dead subgroup (*p* < 0.0001, Figure 12G). The risk scores for patients with tumor free were significantly lower than those for patients with tumor and patients with discrepancy (*p* < 0.0001 and < 0.05, respectively, Figure 12H).

The GSVA result showed that 121 KEGG pathways associated with risk score were significantly enriched (*p* < 0.01, Supplementary Table 7). Thirteen KEGG pathways with a |*cor*| > 0.4 were found to be more active in the high-risk subgroups, including two key OSPs cell cycle (*p* = 0 and *cor* = 0.5975) and P53 signaling pathway (*p* = 0 and *cor* = 0.5202, Figure 12I). There were extremely strong positive correlations between the risk score and the activities of 13 KEGG pathways (all *p* < 0.001, Figure 12J). The GSEA result showed that 17 KEGG pathways were significantly enriched (adjusted *p* < 0.05, Supplementary Table 8). Among these pathways, 9 and 8 KEGG pathways in the high-risk subgroup were significantly upregulated and downregulated, respectively (Supplementary Table 8). Except for upregulated cytokine-cytokine receptor interaction pathway, the other 16 pathways were observed in the pathway list enriched by the GSVA method (Supplementary Tables 7, 8). Top 5 upregulated pathways were cell cycle (adjusted *p* = 1.82e-08), DNA replication (adjusted *p* = 0.001377), homologous recombination (adjusted *p* = 0.023061), oocyte meiosis (adjusted *p* = 0.004126) and P53 signaling pathway (adjusted *p* = 0.023061) (Figure 12K), and the top 5 downregulated pathways were alpha-linolenic acid metabolism (adjusted *p* = 0.030427), cell adhesion molecules (CAMs, adjusted *p* = 0.030427), Fc epsilon RI signaling pathway (adjusted *p* = 0.030427), long-term depression (adjusted *p* = 0.030855) and viral myocarditis (adjusted *p* = 0.022938) (Figure 12L).

### Risk score is an independent prognostic factor in LUAD

**Figure 13:**
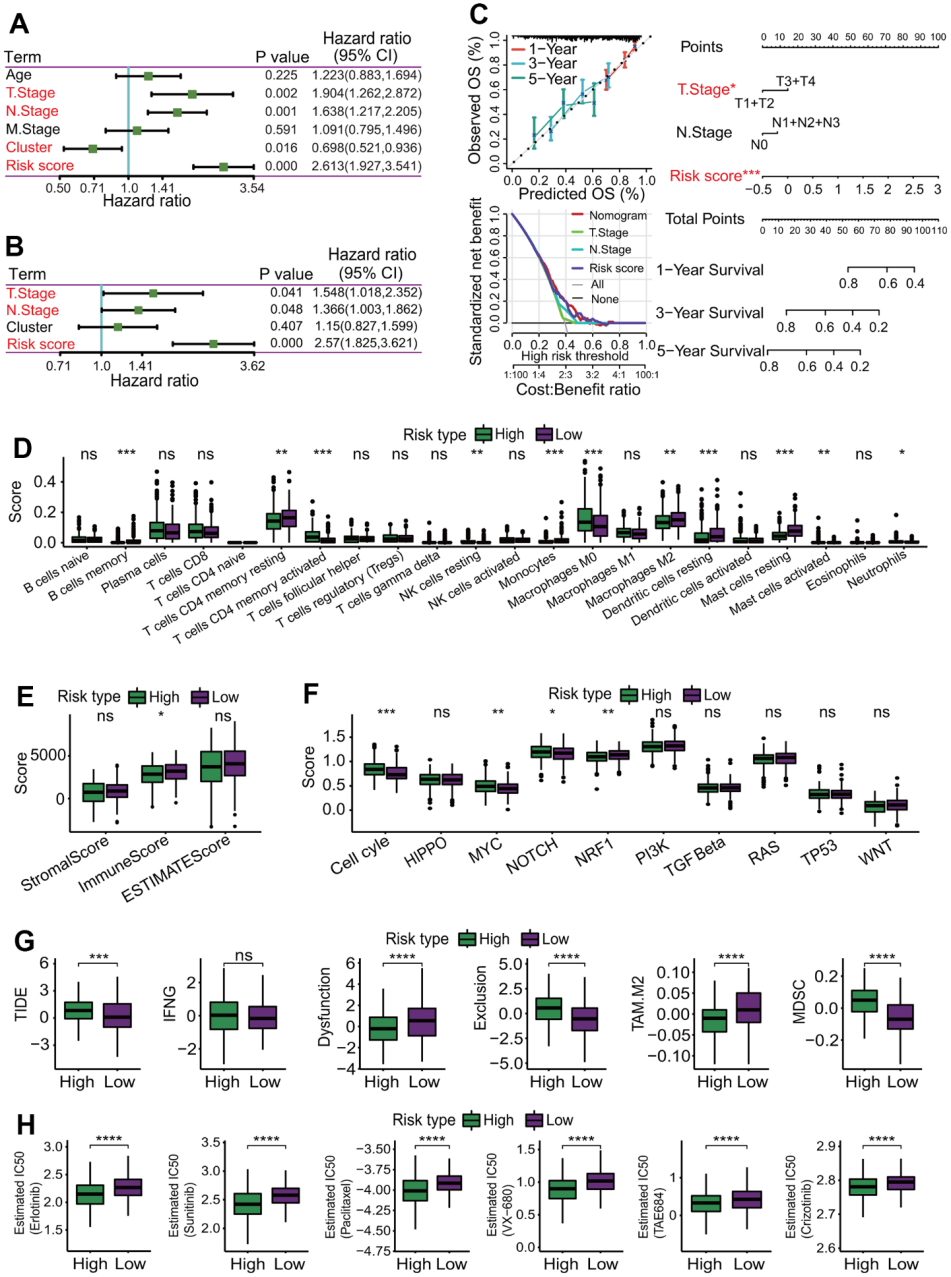
**Independent prognostic analysis and correlations of risk score with immunological features.** (**A**) Independent prognostic analysis for risk score using univariate Cox regression analysis. Risk score was significantly correlated with the overall survival of LUAD patients, and could serve as an independent prognosticator in predicting the survival of LUAD patients. (**B**) Independent prognostic analysis for risk score using multivariate Cox regression analysis. Risk score was significantly correlated with the overall survival of LUAD patients, and could serve as an independent prognosticator in predicting the survival of LUAD patients. (**C**) Construction of a nomogram predicting the survival. A nomogram for predicting 1-year, 3-year and 5-year overall survival was constructed. (**D**) Comparisons of 22 immune cell types between high- and low-risk subgroups. Some immune cell types had significant differences between two risk subgroups. *p < 0.05, **p < 0.01 and ***p < 0.001. (**E**) Comparison of immune infiltration score. LUAD patients in the low-risk subgroup had higher immune scores than those in the high-risk subgroup. *p < 0.05. (**F**) Comparisons of activities of 10 oncogenic signaling pathways between two risk subgroups. The activities of cell cycle, MYC, NOTCH and NRF1 pathways had significant differences, and three oncogenic signaling pathways (cell cycle, MYC, NOTCH) had higher activities in the high-risk subgroup. *p < 0.05, **p < 0.01 and ***p < 0.001. (**G**) Comparisons of tumor immune dysfunction and exclusion (TIDE) score, interferon-gamma (IFNG), T cell dysfunction (Dysfunction), T cell exclusion (Exclusion), tumor-associated macrophages M2 (TAM.M2) and myeloid-derived suppressor cells (MDSC). Except IFNG, those terms had significant differences between two risk subgroups, and TIDE, Exclusion and MDSC in the high-risk subgroup were significantly higher than those in the low-risk subgroup. Dysfunction and TAM. M2 in the high-risk subgroup were significantly lower than those in the low-risk subgroup. *p < 0.05, **p < 0.01, ***p < 0.001 and ****p < 0.0001. (**H**) Comparisons of estimated IC50 values for 6 conventional chemotherapy agents. The estimated IC50 values for 6 chemotherapy agents including erlotinib, sunitinib, paclitaxel, VX-680, TAE684 and crizotinib in the high-risk subgroup were significantly lower than those in the low-risk subgroup. *p < 0.05, **p < 0.01, ***p < 0.001 and ****p < 0.0001.

To evaluate the independent predictive capability of six-gene signature in predicting the survival of LUAD patients, an independent prognostic analysis was performed. A UCRA-based analysis showed that the risk score (*p* = 0.000, HR = 2.631, 95% CI = 1.927-3.541), cluster (subtype) (*p* = 0.016, HR = 0.698, 95% CI = 0.521-0.936), T staging (*p* = 0.002, HR = 1.904, 95% CI = 1.262-2.872) and N staging (*p* = 0.001, HR = 1.638, 95% CI = 1.217-2.205) had significant prognostic values (Figure 13A). Furthermore, a MCRA-based analysis showed risk score (*p* = 0.000, HR = 2.57, 95% CI = 1.825-3.621), T staging (*p* = 0.041, HR = 1.548, 95% CI = 1.018-2.352) and N staging (*p* = 0.048, HR = 1.366, 95% CI = 1.033-1.862) had significant prognostic values (Figure 13B).

To better assess the survival of LUAD patients by incorporating various prognostic factors, a nomogram including the risk score, T staging and N staging was constructed to predict the 1-year, 3-year and 5-year OS rate of LUAD patients (Figure 13C). The calibration curve showed that the actual OS rate was basically in line with the predicted value (Figure 13C). The decision curve showed that the nomogram model had a better predictive ability in predicting the survival of LUAD patients (Figure 13C).

### Risk score was correlated with immunological features in LUAD

To investigate the correlations of the risk score with immunological features, a comprehensive immunological analysis was performed. A CIBERSORT-based analysis showed that the relative abundances of 11 of 22 immune cell types had significant differences between the high- and low-risk subgroups (*p* < 0.05 or < 0.01 or < 0.001, Figure 13D). Among these 11 immune cell types, the abundances of 6 immune cell types including B cells memory (*p* < 0.001), T cells CD4 memory resting (*p* < 0.01), monocytes (*p* < 0.001), macrophages M2 (*p* < 0.001), dendritic cells resting (*p* < 0.001) and mast cells resting (*p* < 0.001) were significantly higher in the low-risk subgroup than those in the high-risk subgroup (Figure 13D). Inversely, the abundances of 5 immune cell types including T cells CD4 memory activated (*p* < 0.001), NK cells resting (*p* < 0.01), macrophages M0 (*p* < 0.001), mast cells activated (*p* < 0.01) and neutrophils (*p* < 0.05) were significantly lower in the low-risk subgroup than those in the high-risk subgroup (Figure 13D). The ESTIMATE result showed that the immune score in the low-risk subgroup was significantly higher than that in the high-risk subgroup (*p* < 0.05, Figure 13E) but no significant differences in the stromal score and ESTIMATE score (both *p* > 0.05, Figure 13E). The activities of 4 of 10 OSPs were significantly different, including cell cycle, MYC, NOTCH and NRF1 (Figure 13F). Among the four OSPs, three OSPs including cell cycle (*p* < 0.001), MYC (*p* < 0.01) and NOTCH (*p* < 0.05) in the high-risk subgroup were more active than those in the low-risk subgroup, and one OSP NRF1 (*p* < 0.01) in the low-risk subgroup was more active than that in the high-risk subgroup (Figure 13F). The TIDE result showed that the TIDE score (*p* < 0.001), exclusion score (*p* < 0.0001) and MDSC score (*p* < 0.0001) were significantly higher in the high-risk subgroup, and the dysfunction score (*p* < 0.0001) and TAM.M2 score (*p* < 0.0001) were significant higher in the low-risk subgroup (Figure 13G). The estimated IC50 values for 6 conventional chemotherapy agents including erlotinib, sunitinib, paclitaxel, VX-680, TAE684 and crizotinib in the high-risk subgroup were significantly lower than those in the low-risk subgroup (all *p* < 0.0001, Figure 13H).

### Quantitative real-time PCR validated the expression of prognostic genes

**Figure 14:**
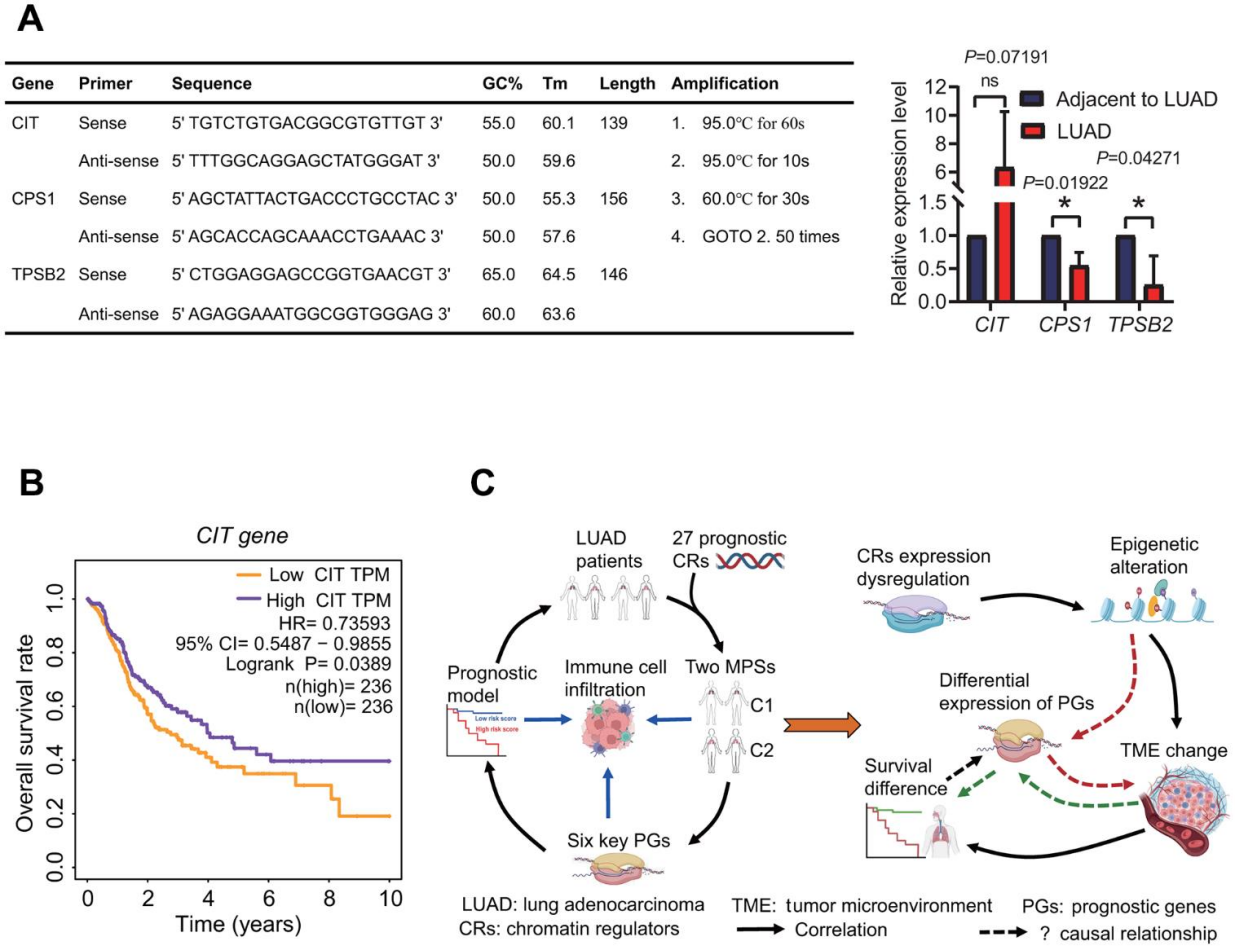
**Expression of prognostic genes and underlying prognostic mechanism.** (**A**) Expression of prognostic genes. *CPS1* and *TPSB2* genes were lowly expressed in LUAD tissues by a quantitative real-time PCR method. (**B**) Survival curve. The high expression of *CIT* gene resulted in a better overall survival rate in patients with lung adenocarcinoma. (**C**) Prognostic results and potential prognostic mechanism based on prognostic chromatin regulators. According to the prognostic results and existing literature, the potential prognostic mechanism was delineated.

To validate the expression of prognostic genes between LUAD and normal lung tissues, three prognostic genes including *CIT*, *CPS1* and *TPSB2* were selected to perform the expression analysis using quantitative real-time PCR (qPCR) method. The result showed that the expression of three genes had a consistent trend with RNA-seq result (Figures 3, 8H, 14A).

## DISCUSSION

Developing a robust prognostic signature is very important to effectively predict the survival of LUAD patients. In recent years, many types of molecular prognostic signatures have been developed, including protein, mRNA, miRNA and lncRNA [6–10]. Some signatures have also showed better predictive capability [38, 39, 42, 45]. However, the high heterogeneity of LUAD suggests that some new prognostic subtypes and signatures must be developed to refine the risk stratification. CRs, as the key participators and even drivers in tumorigenesis, have been shown to have the potential as a robust prognostic signature in some cancers, such as bladder cancer and hepatocellular carcinoma [46, 47]. However, little is known about the roles of CRs in LUAD biology and few studies have investigated the prognostic role in LUAD by a comprehensive analysis. In this study, we first constructed and evaluated two MPSs based on CRs, and established and assessed a six-gene prognostic signature based on two MPSs. Subsequently, the correlations of MPS, key prognostic DEGs and risk score with TICs were systematically investigated. Last, the correlations of MPS and risk score with immunotherapeutic and chemotherapeutic responses were predicted.

Through a comprehensive analysis, 27 DECRs were identified to have association with the survival of LUAD patients and used to construct MPSs. Among these 27 prognostic DECRs, 23 risk and 4 protective CRs were identified. At present, many of these 27 prognostic CRs we identified have been shown to have correlations with prognosis in some cancers [48–52]. For example, *CBX6* and *CBX7* were identified as prognostic biomarkers in bladder cancer [48]. *PBK* was found to be associated with glioblastoma and oral squamous cell carcinoma treated with radiotherapy [49, 50]. *TTK* was identified as a prognostic biomarker in NSCLC [51]. *BUB1* was found as a prognostic factor for hepatocellular carcinoma [52]. In addition, some new prognostic CRs have also been identified in LUAD, such as *NPAS2* and *HMGA2* [53, 54]. In this study, an interesting finding is that *CIT* was highly expressed as a protective gene in LUAD tissue, and the high expression of *CIT* was significantly associated with a high OS rate (Figure 14B). *CIT* was further found to be significantly overexpressed in the C2 subtype with a high OS rate (Figures 4, 5A). Despite this, we have a little confusion about the correlation between the *CIT* expression and the OS in LUAD patients. *CIT* (Citron Rho-Interacting Serine/Threonine Kinase) is a gene encoding a serine/threonine-protein kinase that functions in cell division. Several recent studies have reported the potential role of *CIT* gene in the initiation and progression of some tumors [55–57]. The upregulation of *CIT* could promote the growth of cancer cells [56, 57], and was associated with a poor outcome in some cancers such as bladder cancer and pancreatic ductal adenocarcinomas [58, 59]. On the contrary, some studies showed that the upregulation of *CIT* resulted in a better time to progression in some other cancers such as ovarian carcinomas [60]. From the above, we speculated that whether *CIT* is a risk gene or a protective gene might be related to cancer type. In this study, although we identified that *CIT* was a protective gene in LUAD, the prognostic mechanism still needs to be further revealed.

In the six-gene prognostic signature, *CENPK* (Centromere Protein K) is a subunit of *CENP-H-I* complex that maintains the proper kinetochore function and mitotic progression [61]. In the last five years, the role of *CENPK* has been gradually discovered in cancers and become a research hotspot. A few recent studies have reported that *CENPK* played roles in cervical cancer, thyroid cancer, ovarian cancer, etc. [62–64]. The overexpression of *CENPK* promoted cell proliferation and migration that was correlated with a poor prognosis [63, 64], and the expression downregulation of *CENPK* suppressed cell growth and inhibited cancer progression [65, 66]. So far, only a few studies were found to report that *CENPK* was upregulated in LUAD, and the overexpression of *CENPK* was associated with advanced LUAD and poor prognosis of LUAD patients [67]. Our result was consistent with those of the above findings, which further confirmed the prognostic role of *CENPK* in LUAD. *ANGPTL4* (Angiopoietin like 4) encodes a glycosylated protein that plays roles in regulating lipid metabolism, glucose homeostasis and insulin sensitivity. In some cancers, the *ANGPTL4* expression promoted venous invasion and cancer progression in breast cancer, thyroid cancer, colorectal cancer and gastric cancer [68–71], and was associated with a poor prognosis in breast cancer [68]. In addition, some studies have also showed that *ANGPTL4* had tumor-suppressive role, even dual roles in some cancers such as urothelial carcinoma [72]. So far, little is known about the roles of *ANGPTL4* in LUAD. Our result showed that *ANGPTL4* was significantly upregulated in LUAD and was associated with a poor prognosis. *CPS1* (Carbamoyl-Phosphate Synthase 1) encodes a crucial mitochondrial enzyme that catalyzes the synthesis of carbamoyl phosphate in the urea cycle. Several recent studies showed the associations of *CPS1* with some cancers such as gastric cancer and ovarian cancer [73, 74]. Two recent published studies showed that the expression level of *CPS1* was upregulated in LUAD tissue, and its high expression resulted in a poor survival rate [75, 76]. Some additional studies showed that lncRNA *CPS1* intronic transcript 1 (*CPS1-IT1*) as a positive regulatory factor suppressed cell invasion and metastasis in colorectal cancer, ovarian cancer and LC [77–79]. The above studies indicated that *CPS1* as a risk factor might play an important role and serve as a potential prognostic marker in LUAD. Our result was consistent with the above results, which supports the opinion of *CPS1* as a potential prognosticator. *CCL20* (C-C Motif Chemokine Ligand 20), belonging to the subfamily of small cytokine CC genes, plays important roles in the chemotaxis of some immune cells including dendritic cells, effector/memory T cells and B cells by the ligand-receptor pair CCL20-CCR6. Some recent published studies have demonstrated that *CCL20* promoted cancer progression by some pathways such as by activating p38 pathway in laryngeal cancer [80], through PI3K pathway in LC and via NF-kappa B pathway in thyroid cancer [81, 82]. *GJB3* (Gap Junction Protein Beta 3) belonging to the connexin gene family, encodes the Connexin 31. The mutations of *GJB3* are mainly associated with non-syndromic deafness or erythrokeratodermia variabilis. To date, only a recent study was found to report that *GJB3* as a predictor of a 9-mRNA prognostic signature was associated with the survival of LUAD patients and was identified as a protective factor [83]. What’s different was that our result showed that *GJB3* was a risk gene in the 6-mRNA prognostic signature we identified. The reasons for the difference remain unclear, which forces us to explore more deeply the association of *GJB3* with LUAD in the future. *TPSB2* (Tryptase Beta 2) is the only protective factor in the prognostic signature established in this study. *TPSB2* encoding the beta-tryptase, is mainly expressed in mast cells and is a common marker for mast cell. Several previous studies showed that *TPSB2* was abnormally expressed or somatic genomic alterations occurred in some cancers including breast cancer and gastric cancer [84, 85]. A growing evidence now shows that *TPSB2* expression is a potential prognostic marker in cancers including gastric cancer [85].

Notably, the gene expressions in the six-gene signature were significantly correlated with immune cell infiltration. Especially, the *CPS1* expression was strongly negatively correlated with immune cell infiltration, while the *TPSB2* expression was strongly positively correlated with immune cell infiltration. Furthermore, the risk score based on the six-gene signature was significantly correlated with immunological status of LUAD patients. It is well known that tumor immune microenvironment (TIME) plays important roles in cancer biology, and patients with differing TIME present differing immunotherapeutic responses and clinical outcomes [86–88]. Tumor immunophenotype has been gradually recognized as an independent prognostic factor to estimate the prognosis [89]. As a whole, tumor patients with higher immune infiltration levels had a higher OS rate [90, 91]. In this study, LUAD patients in the low-risk subgroup had lower risk scores and higher infiltration levels of some immune cell types including B cells memory, mast cells resting, dendritic cells resting, which was consistent with the results from previous published researches [90–92]. From the above researches, we speculate that the abnormal expression of six prognostic genes may have an influence on immune cell infiltration or immune infiltration affects their expression. However, we still do not know the potential causal relationship between them by reviewing a large amount of literature. Despite the important role of TIME in tumor biology, the potential correlations of immune cells infiltration with the prognosis remain ambiguous. Even the associations between the infiltration levels of some immune cells with the prognosis are paradoxical in different studies or subpopulations. For example, the correlations of increased mast cells with either good or poor prognosis depended on some factors such as tumor type and stage [93]. So, it is important to reveal the underlying prognostic mechanism of prognostic signature, which not only contributes to explaining the biological mechanism of cancer development and progression, but also offers a great help for evaluating the survival of tumor patients more accurately. In view of this, we summarized the results to delineate the underlying relationship between CRs, six-gene prognostic model, immunological features and the prognosis (Figure 14C). Now, it is clear that the epigenetics regulation and tumor microenvironment are two important factors affecting tumor formation and development, and there is a complex interactive relationship among cancer, epigenetics and immunity. CRs as indispensable upstream regulatory factors of epigenetics, drive the epigenetics alterations by acting as “writers”, “readers”, “erasers” of histone and DNA, or “remodelers” of chromatin. CRs have been shown to play key roles in driving cancer by numerous studies [16–19]. In this study, we hypothesize that the expression dysregulation of 27 CRs causes the epigenetic alteration in LUAD patients, which results in the TME changes. Different TMEs led to the differences in the survival and the expression of six prognostic genes in LUAD patients. Of course, the underlying prognostic mechanism must be verified on the basis of the correlations of the expressions between these genes, and of their expressions with immune cell infiltration and the OS rate of LUAD patients in the future.

In addition, a lot of signaling pathways were identified to have higher activities in LUAD patients with a poor prognosis by the GSAV and GSEA methods, including cell cycle and p53 signaling pathways. At present, a large number of studies have demonstrated that the dysregulated activity of many signaling pathways was closely associated with the occurrence and progression of tumor, and the landscape of pathway alterations in 33 cancer types have been charted in detail, including LUAD [94]. As far as we know, the cell cycle plays critical role in the regulation of mitotic cell cycle progression [95], and its alterations were the most common in many tumors, except for rare alterations in uveal melanoma, thymoma, testicular cancer and acute myeloid leukemia [94]. Some genes in the cell cycle have been identified to be associated with LC, and serve as potential prognostic markers including *BUB1B*, *ZWINT*, *CDC20*, etc. [96–98]. The high expression of these genes was often associated with a poor prognosis in some cancers including LUAD [96, 99–102]. Like the cell cycle, the p53 signaling pathway is one of the most common oncogenic pathways across various cancers [94], and one of its key functions is to regulate the cell cycle [103]. In fact, the p53 signaling pathway is connected to its downstream pathway cell cycle (https://www.kegg.jp/kegg/pathway.html). The high activities of the cell cycle and p53 pathways were associated with a worse prognosis in LUAD patients, indicating that these two signaling pathways contribute to LUAD progression. In addition, some pathways involved in the replication and repair were identified to be negatively correlated with the survival of LUAD patients, showing that the upregulation of these pathways are identical to the upregulation of the cell cycle and p53 signaling pathways. Currently, some therapeutic approaches targeting signaling pathways have been investigating or have been approved for therapy, such as cell cycle, PI3K, WNT, RAS and p53 signaling pathways [94]. And some therapeutic targets have been investigating and new drugs against these targets are being designed, such as cyclin-dependent kinases (CDKs) [104]. Understanding the alterations is critical for the development of new therapeutic approaches in the oncogenic signaling pathways, which will contribute to improve the care of LUAD patients. Meanwhile, the identification of oncogenic signaling pathways related to the survival will provide a valuable resource for LUAD precision medicine.

Although a six-gene prognostic signature was established based on MPSs constructed by CRs and the validity of MPSs and the robustness of the prognostic model have been well evaluated using some other independent datasets, some limitations should be mentioned. First, the current study was a retrospective study and required further validation in prospective clinical studies. Second, the underlying mechanism by which CRs predict the survival of LUAD patients through six prognostic genes needs further investigation.

## CONCLUSIONS

In conclusion, the current study developed two MPSs based on CRs and established a new six-gene prognostic signature based on MPSs in LUAD. The MPS and risk score were significantly correlated with the TIME. These findings provide novel insights into the biological mechanism of CRs in LUAD biology, which may offer a help for building a more accurate prognosis assessment system and personalized immunotherapy system.

## MATERIALS AND METHODS

### Collection of samples and clinical data

The gene expression datasets and clinical data for LUAD were retrieved from the public databases The Cancer Genome Atlas (TCGA, https://portal.gdc.cancer.gov/) and Gene Expression Omnibus (GEO, https://www.ncbi.nlm.nih.gov/geo/). The TCGA-LUAD dataset was used to construct the MPS and prognostic signature. The GEO-LUAD datasets including GSE31210, GSE30219, GSE37745, GSE50081, GSE42127, GSE3141, GSE26939, GSE29016, GSE68465, GSE72094 and GSE19804 were used to test the validity of MPS and the robustness of prognostic model. The main information for these datasets was listed in Table 2, including sample number, data platform, gender and survival status. All datasets have been approved by the Institutional Review Board of relevant participating institutions and no additional approval was required in this study.

**Table 2:** Main clinical information of datasets in this study.

**Datasets**	**Platform**	**Number**	**Gender**			**Age**			**Stage**			**Survival status**		**Application**
			**Female**	**Male**		**<=60**	**>60**		**I and II**	**III and IV**		**Alive**	**Dead**	
TCGA	HT-seq	472	255	217		146	326		378	86(8?)		289	183	Model construction
GSE31210	GPL570	226	121	105		108	118		226	0		191	35	Model evaluation
GSE30219	GPL570	85	19	66		43	42		/	/		40	45	Model evaluation
GSE37745	GPL570	106	60	46		46	60		89	17		29	77	Model evaluation
GSE50081	GPL570	129	62	67		19	110		62	67		76	53	Model evaluation
GSE42127	GPL6884	133	65	68		38	95		111	22		90	43	Model evaluation
GSE3141	GPL570	111	/	/		/	/		/	/		52	59	Model evaluation
GSE26939	GPL9503	116	51	65(16?)		40	76		71	45(30?)		49	66(1?)	Model evaluation
GSE29016	GPL6947	39	21	18		12	27		36	3(1?)		10	29	Model evaluation
GSE68465	GPL96	443	220	223		147	296		/	/		207	236	Model evaluation
GSE72094	GPL15048	442	240	202		70	372(21?)		334	108(28?)		298	144(22?)	Model evaluation
GSE19804	GPL570	60 pairs	60	0		29	31		47	13		/	/	Expression analysis
GEPIA database	HT-seq	820	/	/		/	/		/	/		/	/	Expression analysis
Real sequencing data	Hiseq2000	10 pairs	5	5		10	0		10	0		/	/	Expression analysis

### Production of transcriptome sequencing data

The transcriptomes of LUAD and normal lung tissues from 10 LUAD patients (5 male and 5 female) of 35-50 years old were sequenced using the Illumina sequencing platform with the paired-end method by Beijing Novogene Technology Co., Ltd. (China). All tissues were collected by The First People’s Hospital of Yunnan Province and informed consent was obtained from all LUAD patients. This study was approved by the Institutional Review Board of The First People’s Hospital of Yunnan Province (No. 2017YY227). The transcriptome sequencing data were used to evaluate the expression levels of prognostic genes between LUAD and normal lung tissues.

### Acquisition of gene set

Totals of 870 CRs and 10 oncogenic signaling pathways (OSPs) were downloaded from the previous studies by Lu et al. [15] and by Sanchez-Vega et al. [94], respectively.

### Identification of differentially expressed gene and functional analysis

Differentially expressed genes (DEGs) were identified using differentially expressed gene analysis (DEGA) based on the limma package (version 3.52.2) in the Bioconductor project (version 3.15, http://www.bioconductor.org/) [105]. An empirical Bayes (eBayes) method was used to assess the gene expression change by a moderated t-test. The Benjamini-Hochberg correction was used to adjust *p*-value for multiple testing.

Functional analysis based on KEGG pathway set was implemented using the gene set variation analysis (GSVA) method in the GSVA package (version 1.44.2) [106] and the gene set enrichment analysis (GSEA) method in the clusterProfiler package (version 3.14.3) [107], respectively. GSVA is a non-parametric and unsupervised gene set enrichment (GSE) method. GSVA estimates pathway activity variation over a sample population by characterizing pathways from a gene expression profiling. The non-parametric kernel estimation of the cumulative density function for each gene expression profile was performed using the Gaussian kernel. The enrichment score for each sample was calculated using GSVA method. The maximum and minimum number of genes was set at 100 and 10 in an output gene set, respectively. GSEA was performed using the default parameters. The maximum and minimum number of genes was set at 500 and 10, respectively. The permutation number was set at 1000 and multiple testing was corrected using the Benjamini-Hochberg method. The Wilcoxon’s test method was used to compare the difference of enrichment pathways between differing subgroups.

### Construction of prognostic model

A univariate Cox regression analysis (UCRA) was applied to identify genes associated with the prognosis using the R survival package (version 3.2.13). The least absolute shrinkage and selection operator (LASSO) Cox regression analysis was used to regularize the genes with significant UCRA prognostic value. The basic idea behind the LASSO algorithm is that an added L1-norm regularization term is used to penalize the weight of variables in a linear model. The LASSO regularization term is defined as:


$$\lambda {\left\|\omega \right\|}_{1}$$

where ${\left\|\omega \right\|}_{1}$ is the L1-norm of the coefficient vector and λ is a control parameter. The optimal λ value is set by the cross-validation and a 10-round cross-validation was performed to prevent the overfitting. The coefficients of some variables with minor contributions are forced to be 0 by the LASSO regularization, which enables the model to generate a sparse variable space. The parameter compression characteristic is vital for feature selection and was widely used to construct the low-complexity prognostic model for risk prediction in cancer patients. LASSO Cox regression analysis was performed using the R glmnet (version 4.1.4) package. A set of genes with the most important prognostic feature was identified by the LASSO method. To establish the optimal prognostic model, a multivariate Cox regression analysis (MCRA) by Akaike information criterion (AIC) was used to optimize the LASSO prognostic genes based on the R MASS package (version 7.3.55). The risk score for each patient was calculated according to the following formula:


$$Risk\;score=\sum_{i}Coe(Gene_{i})*Exp(Gene_{i})$$

where *Coe*(*Gene_i_*) is the MCRA Cox coefficient of the *i* gene and *Exp*(*Gene_i_*) is the mRNA level of the *i* gene. On the basis of the median risk score, LUAD patients were divided into high-risk and low-risk subgroups. The Kaplan-Meier (KM) estimator and log-rank test were used to assess the OS rate of LUAD patients between two risk subgroups based on the R survival package (version 3.2.13). The receiver operating characteristic (ROC) analysis was used to evaluate the predictive capability for prognostic model using the R survivalROC package (version 1.0.3), and the area under curve (AUC) indicated the predictive accuracy of prognostic model in the ROC curve.

### Identification of MPSs based on prognostic CRs

MPSs were identified based on the differentially expressed CRs (DECRs) with the UCRA prognostic value using an unsupervised consensus clustering algorithm. The consensus clustering method is one of the most commonly used methods for classifying cancer subtypes by adopting resampling strategy of different omics data sets. In this study, the partitioning around medoid (PAM) algorithm with the Spearman correlation distance metric was applied to the consensus clustering based on the gene expression profiles of 27 prognostic DECRs from 472 LUAD patients in the TCGA database, and performed 500 bootstraps each 80% resampling of LUAD patients. The unsupervised consensus clustering was performed using the R ConsensusClusterPlus package (version 1.60.0) [108]. The maximum number of clusters (k) is set to 10, and the optimal k value was assessed on the basis of the cumulative distribution function and consensus matrix.

### TICs analysis

First, the immune infiltrations of 22 types of TICs were estimated between differing MPSs and between differing risk subgroups using the CIBERSORT (cell-type identification by estimating relative subsets of RNA transcript) method [109]. On the basis of gene expression profiles, the proportions of 22 types of TICs were quantified using the LM22 signature and 100 permutations by the CIBERSORT algorithm. The relative abundances of 22 types of TICs between differing subgroups were compared using the Wilcoxon rank sum test.

Second, the infiltration of immune and stromal cells between differing MPSs and between differing risk subgroups was assessed using the ESTIMATE (estimation of stromal and immune cells in malignant tumors) algorithm in the R estimate package (version 1.0.13) [110]. The fraction of immune and stromal cells was inferred using the gene expression signatures in LUAD samples and compared using the Wilcoxon rank sum test.

Last, tumor immune estimation resource (TIMER, http://cistrome.org/TIMER/) was used to assess the immune infiltrations of 6 types of TICs including dendritic cells, macrophages, neutrophils, CD8 T cells, CD4 T cells and B cells [111], and the correlations of TICs with risk genes were measured using the Pearson correlation coefficient.

### Prediction of therapeutic response

The potential response of immune checkpoint blockade (ICB) therapy between differing subgroups was predicted using the Tumor Immune Dysfunction and Exclusion (TIDE) algorithm (http://tide.dfci.harvard.edu/) [112]. The TIDE score, interferon-gamma (IFNG) score, T cell dysfunction (Dysfunction) score, T cell exclusion (Exclusion) score, tumor-associated macrophages M2 (TAM.M2) score and myeloid-derived suppressor cells (MDSC) score were used to evaluate potential therapeutic responses. To further investigate potential responses of ICB therapy, the correlations of 44 immune checkpoint genes (ICGs) with risk genes were measured using the Pearson correlation coefficient [113].

Potential clinical chemotherapeutic responses of LUAD patients to conventional chemotherapy agents were predicted using the R pRRophetic package (version 0.5) [114]. The half-maximal inhibitory concentration (IC50) was used to evaluate the drug sensitivity.

### Activity assessment of 10 OSPs

The activities of 10 OSPs including 335 genes between differing MPSs and between differing risk subgroups were assessed using the single sample gene set enrichment analysis (ssGSEA) in the R GSVA package (version 1.44.2) [106]. The 10 OSPs were separate cell cycle (15 genes), HIPPO (38 genes), MYC (13 genes), NOTCH (71 genes), NRF1 (3 genes), PI3K (29 genes), TGF-beta (7 genes), RAS (85 genes), TP53 (6 genes) and WNT (68 genes). A list of 335 genes can be downloaded by referring to a published article [94].

### ssGSEA

The ssGSEA was used to assess the immune cell infiltration in LUAD tissues. A marker gene set for 29 types of immune cells was obtained from a published article [115]. The relative abundances of 29 types of immune cells were quantified using the ssGSEA method in the R GSVA package (version 1.44.2) [106].

### Statistical analysis

All the statistical analyses of the data in this study were performed based on the open-source R software (version 4.1.3). The DEGA was performed to identify DEGs using a moderated t-test method, and a gene with an adjusted *p* < 0.05 and a |log2(FC)| > log2(1.5) was considered to significantly change in expression. The GSVA and GSEA were performed to investigate the function of DEGs using the Wilcoxon’s test method, and an adjusted *p* < 0.05 was considered to have significant functional change. An unsupervised consensus clustering algorithm was applied to identify MPSs. The UCRA was performed to identify genes associated with the prognosis, and a gene with a *p* < 0.05 was considered to have significant association with the survival. The association of prognostic signature with the OS was evaluated using the KM method, and a *p* < 0.05 was considered to have a significant association. Immune cell infiltrations between differing MPSs and between differing risk subgroups were compared using the Wilcoxon’s test method, and a *p* < 0.05 was considered to have a significant difference. The correlations of MPS and risk score with clinical features were compared using a chi-square test method, and a *p* < 0.05 was considered to have a significant correlation. The expression levels of prognostic genes were validated by a paired t test method between LUAD and normal lung tissues using transcriptome sequencing data, and a *p* < 0.05 was considered to have a significant change. The accuracy of transcriptome sequencing data was evaluated using the qPCR method, and the relative expression levels of genes were compared using an unpaired t test method and a *p* < 0.05 was considered to have a significant change in expression.

## Supplementary Material

Supplementary Figures

Supplementary Table 1

Supplementary Table 2

Supplementary Table 3

Supplementary Table 4

Supplementary Table 5

Supplementary Table 6

Supplementary Table 7

Supplementary Table 8
