# Supplementary material for: A robust six-gene prognostic signature based on two prognostic subtypes constructed by chromatin regulators is correlated with immunological features and therapeutic response in lung adenocarcinoma
Source: Aging (Albany NY). 2023 Nov 7;15(21):12330–68. doi: 10.18632/aging.205183 (PMC10683604; doi:10.18632/aging.205183)
Supplement: Supplementary Figures [file aging-15-205183-s001.pdf]

SUPPLEMENTARY FIGURES

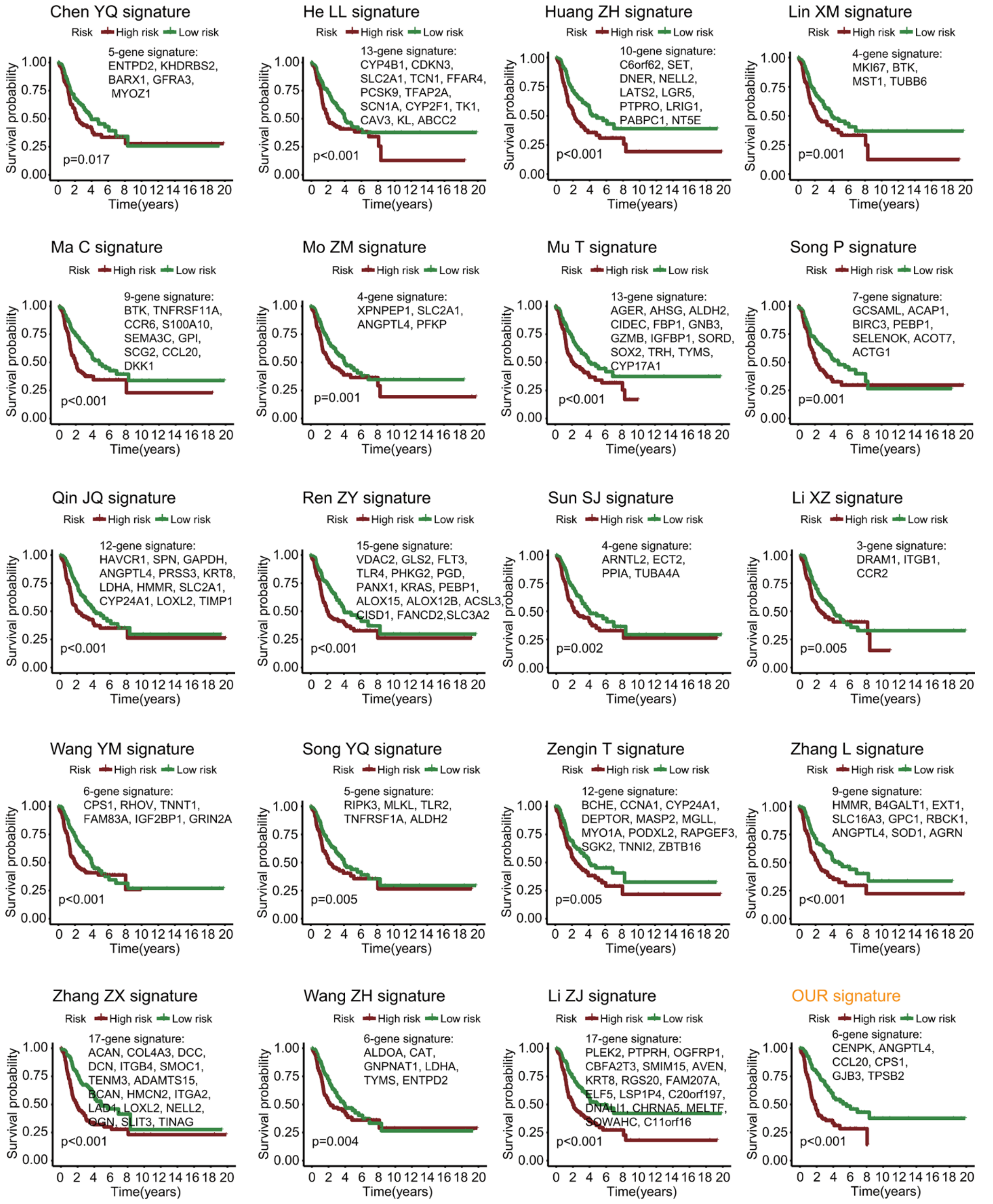

Supplementary Figure 1. Survival curves of 20 prognostic signatures.

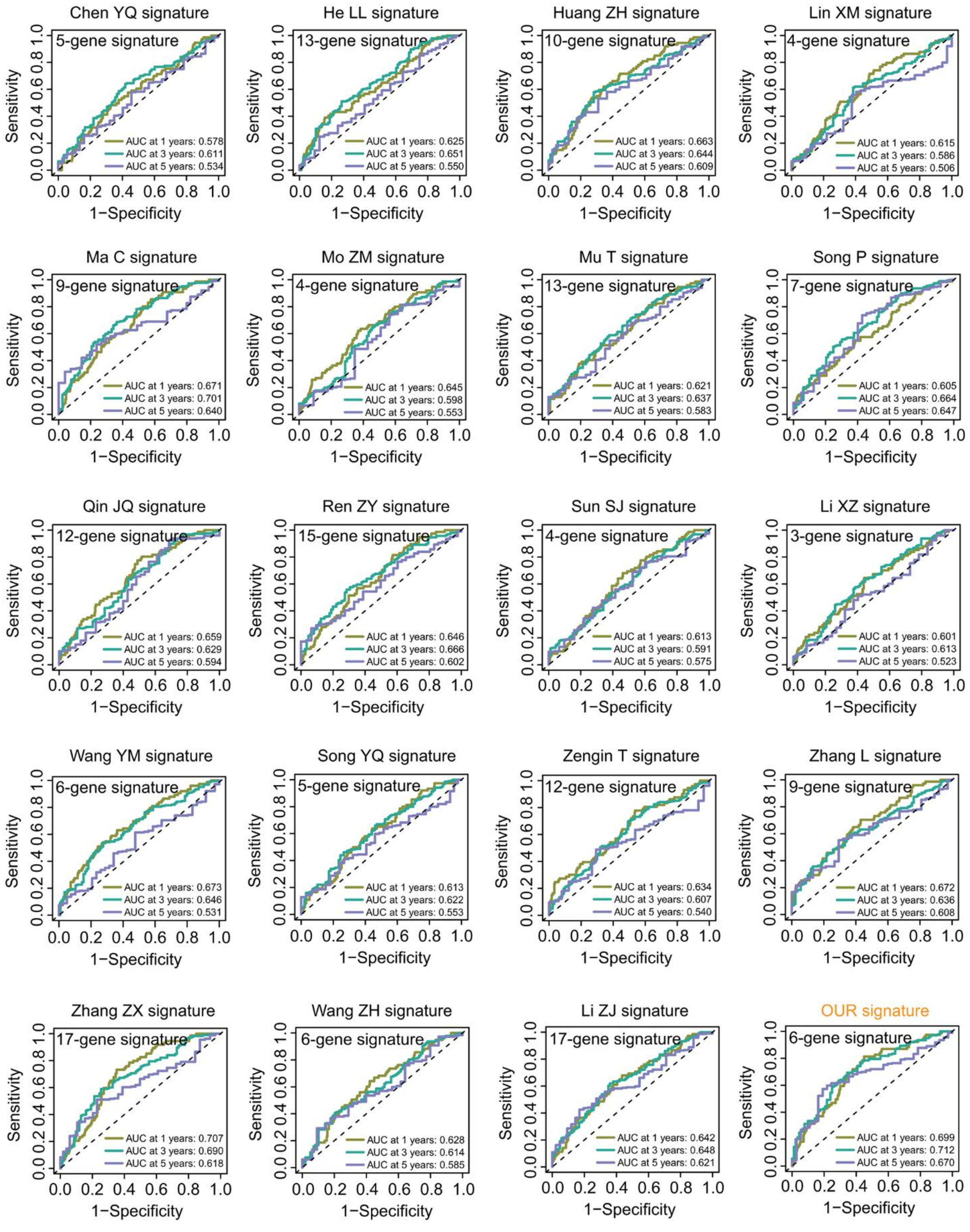

Supplementary Figure 2. Receiver operating characteristic curves of 20 prognostic signatures by weighting aalen method.

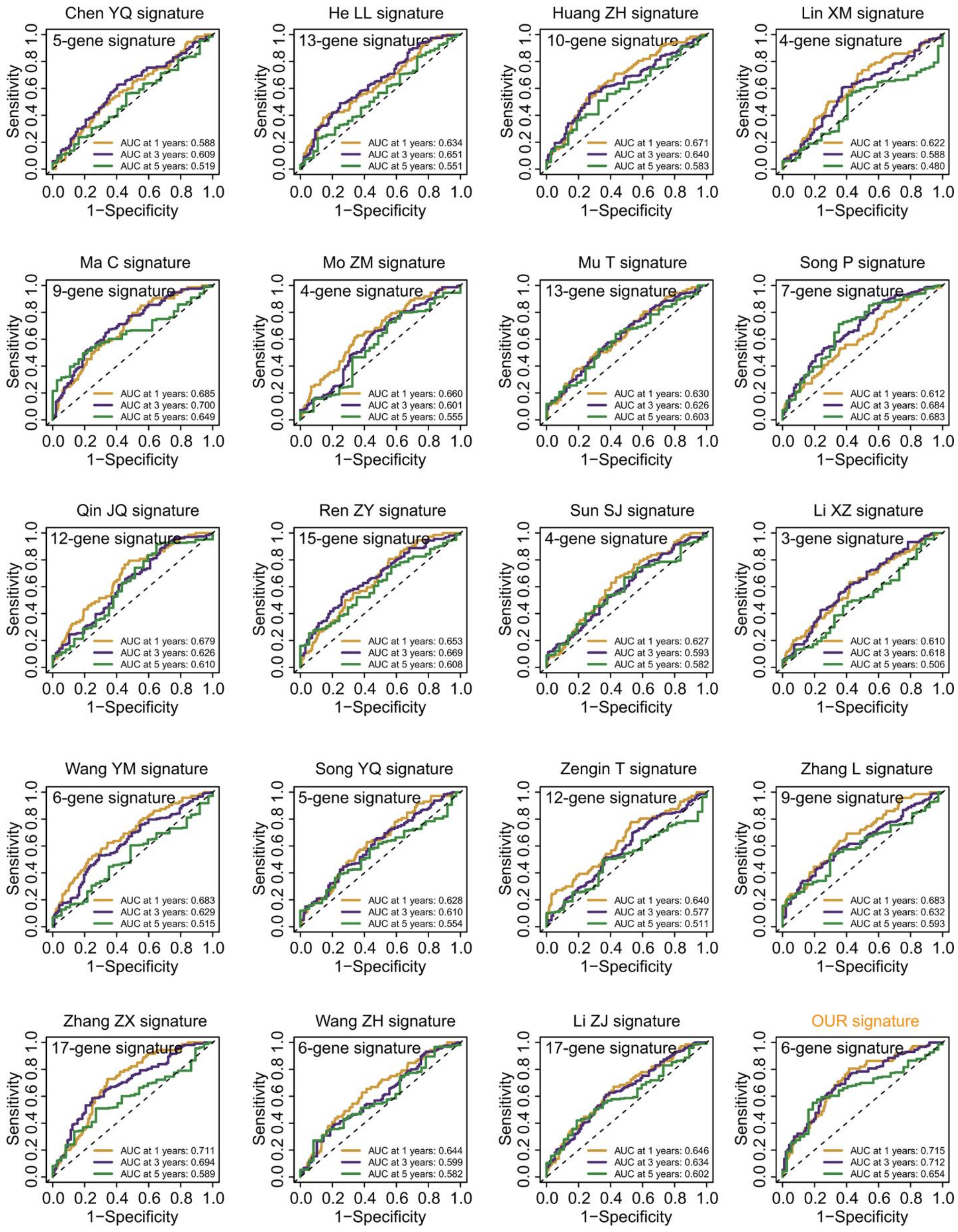

Supplementary Figure 3. Receiver operating characteristic curves of 20 prognostic signatures by weighting marginal method.
