# Supplementary material for: A robust six-gene prognostic signature based on two prognostic subtypes constructed by chromatin regulators is correlated with immunological features and therapeutic response in lung adenocarcinoma
Source: Aging (Albany NY). 2023 Nov 7;15(21):12330–68. doi: 10.18632/aging.205183 (PMC10683604; doi:10.18632/aging.205183)
Supplement: Supplementary Table 8 [file aging-15-205183-s009.pdf]

Supplementary Table 8. KEGG pathways enriched by GSEA method.

Description	setSize	E_Score	NES	p value	p.adjust	q value
**Upregulated pathways**						
DNA Replication	35	0.736449729396368	2.03690969516193	0.0000151369217012898	0.00137745987481737	0.00117908653252152
Homologous Recombination	26	0.724404474641536	1.90935319570508	0.000513905955422847	0.0230609512890726	0.0197398541977024
Mismatch Repair	22	0.721934747964346	1.82178519953093	0.00155816303419186	0.030426628602272	0.0260447717358951
Cell Cycle	119	0.672607485893627	2.27660731776706	0.0000000001	0.0000000182	1.55789473684211E-08
Proteasome	42	0.602774052010137	1.72675095759965	0.00183956842508467	0.030426628602272	0.0260447717358951
P53 Signaling Pathway	65	0.566574926625628	1.76199825945229	0.000633542617831664	0.0230609512890726	0.0197398541977024
Oocyte Meiosis	91	0.548935448235104	1.79946064847862	0.000068003858822793	0.00412556743524944	0.00353142845816609
Pyrimidine Metabolism	89	0.492113129094618	1.60118875704439	0.00200615133641354	0.030426628602272	0.0260447717358951
Cytokine Cytokine Receptor Interaction	188	0.398099737708487	1.45859198111463	0.00448783412482144	0.0480462241598531	0.041126900958116
**Downregulated pathways**						
Cell Adhesion Molecules Cams	109	-0.489168465420973	-1.62245237410999	0.00144964281019493	0.030426628602272	0.0260447717358951
Fc Epsilon Ri Signaling Pathway	69	-0.55595778141551	-1.69713254845712	0.00149733080740598	0.030426628602272	0.0260447717358951
Long Term Depression	52	-0.576665210604918	-1.66761252448888	0.00220390715752221	0.0308547002053109	0.0264111950860961
Viral Myocarditis	57	-0.588035222535862	-1.74220768693252	0.000986954262565194	0.0299376126311442	0.02562618085257
Valine Leucine and Isoleucine Degradation	43	-0.58869102821266	-1.65698112512393	0.00417156420719961	0.0474515428568956	0.0406178620174699
Arachidonic Acid Metabolism	41	-0.596886153027357	-1.66240044710594	0.00350612041001675	0.0455795653302178	0.0390154752392842
Asthma	20	-0.718605219525618	-1.71889016635074	0.00395824541300939	0.0474515428568956	0.0406178620174699
Alpha Linolenic Acid Metabolism	15	-0.76076493050057	-1.71769231258579	0.00198981558384615	0.030426628602272	0.0260447717358951
